# *Mx1*-labeled pulp progenitor cells are the main contributors of odontoblast and dentin regeneration in murine molars

**DOI:** 10.1038/s12276-025-01511-3

**Published:** 2025-08-13

**Authors:** Dongwook Yang, Youngjae Jeong, Laura Ortinau, Jea Giezl Solidum, Dongsu Park

**Affiliations:** 1 Department of Molecular Human Genetics, Baylor College of Medicine, Houston, TX USA; 2 Department of Biochemistry and Molecular Biology, College of Medicine, University of the Philippines, Manila, Philippines; 3 Department of Pathology and Immunology, Baylor College of Medicine, Houston, TX USA; 4 Center for Skeletal Biology, Baylor College of Medicine, Houston, TX USA

**Keywords:** Stem-cell research, Regeneration

## Abstract

Regeneration of dentin and odontoblasts from dental pulp progenitor cells is essential for the maintenance of permanent tooth. However, the in vivo identity of endogenous pulp progenitor cells and how they contribute to reparative dentinogenesis remain elusive. Here we show that comparative single-cell analysis of pulp cells before and after molar eruption reveal that endogenous pulp progenitor cells are enriched in coronal papilla-like cells with *Mx1-Cre* and *Cxcl12–*GFP expression. Further, lineage tracing and fluorescence-activated cell sorting analysis indicated that *Mx1-*labeled (*Mx1*^+^) pulp cells include long-term repopulating progenitor cells with higher expression of stem cell markers. Notably, these *Mx1*^+^ progenitor cells contribute to the majority of pulp cells and new odontoblast-like cells in the loaded plane of the molar after eruption. Upon molar injury, *Mx1*^+^ progenitor cells localize into the injury site and differentiate into new odontoblast-like cells, forming *osteocalcin*–GFP^+^ and *scleraxis*–GFP^+^ processes to reoccupy existing dentinal tubules and reparative dentin formation. Taken together, our findings demonstrate that *Mx1* labels dental pulp progenitor cells, which are the major source of pulp cells and odontoblast-like cells with reparative dentinogenesis in vivo.

## Mx1 labels key progenitor cells for tooth regeneration

Teeth contain special pulp cells that can become odontoblasts, which produce mineralized dentin. However, their identity and mechanisms for odontoblast regeneration were unknown. This study explores which pulp cells help repair damaged dentin and maintain odontoblasts in adult teeth. Researchers used comparative single-cell analysis of pulp cells and identified a group of pulp progenitor cells are marked by a gene called *Mx1*. Further, they developed special fluorescent mice that highlight these progenitor cells and tracked how these cells behave over time and after tooth injury. The study found that *Mx1*-marked progenitor cells are crucial for forming new odontoblasts, repairing dentin after tooth damage and maintaining healthy teeth as they age. The findings suggest that understanding these pulp progenitor cells could lead to better ways to treat tooth damage and improve dental health in the future.

This summary was initially drafted using artificial intelligence, then revised and fact-checked by the author.

## Introduction

All human teeth are permanent after the replacement of deciduous teeth and, therefore, the natural regeneration and repair of tooth cells and mineral layers are essential for the long-term maintenance of dental tissues. In particular, dental pulp cells and differentiated dentin-producing odontoblasts play an important role in supporting tooth structure, absorbing the mechanical pressure from mastication and sensing thermal, osmotic and tactile stimuli^1^. When a tooth is exposed to wear, malignant caries, and fractures, the pulp and odontoblasts contribute to the development of a protective barrier, dentin regeneration, and immune responses. By contrast, severe pulpal damage or endodontic treatment can cause loss of sensation, increased fragility, and tooth loss. Previously, external stimuli such as uniaxial tensional/compressional stress have been reported to induce the proliferation of pulp progenitor cells and odontogenic differentiation^2–5^. Furthermore, a recent odontoblast depletion study showed that new odontoblast-like cells can be regenerated from the cell-rich zone of the pulp, suggesting the presence of progenitor cells in adult molar pulp^6^. Therefore, it is likely that proper maintenance and regulation of dental pulp progenitor cells and their differentiation to odontoblast-like cells are important for permanent tooth health and maintenance. However, due to the heterogeneity of pulp cells and the limited availability of animal models to label endogenous pulp progenitor cells, it is largely unknown which cells are dental pulp stem/progenitor cells and how they contribute to endgogenous odontoblast regeneration.

During tooth development, *Wnt1*-Cre^+^ neural crest cells are a known source of primitive pulp (papilla) cells and odontoblasts for primary and secondary dentin formation^7–9^. However, in permanent teeth, pulp cells near odontoblasts proliferate and differentiate into odontoblast-like cells in response to odontoblast depletion^6,8,9^. Similarly, *Axin2*^+^ cells or *aSMA*^+^ perivascular cells in the dental pulp expand through proliferation and gave rise to odontoblast-like cells^10,11^. By contrast, lineage tracing of *Sox10*-CreER^+^ and *PLP*-CreER^+^ cells with a Schwann cell origin showed that glial cells generate pulp progenitor cells and contribute to odontoblasts in adult growing incisors^12^. Furthermore, recent studies revealed that *Gli1*^+^ cells and PTHrP^+^ cells in the developmental tooth apical region give rise to periodontal ligaments (PDLs), alveolar bone and a subset of pulp cells but do not contribute to postnatal pulp cells and odontoblasts, indicating the presence of a heterogeneous pulp progenitor population^13–15^. In fact, many of these studies used developmental or tooth injury models that can cause pulp exposure and the destruction of adjacent dentin structures. More importantly, tooth environments before and after eruption are very different. The tooth before eruption has limited external mechanical or microbial stimuli^16^ and the tooth roots (the apical ends) are not fully developed. However, an erupted tooth is exposed to dynamic environmental factors such as mastication forces^5,17^ with permanent enamel layers and root formation. These posteruptive changes substantially impact the function of inner pulp cells and odontoblasts^17,18^. Therefore, it is still not clear whether existing preodontoblasts or new odontoblast-like cells from pulp progenitor cells replace injured odontoblasts during the regeneration process. Moreover, it is essentially unknown which of the pulp cells are long-term repopulating progenitor cells and whether they contribute to the recycling of adult pulp cells and odontoblasts in permanent teeth.

In this study, we sought to define the molecular characteristics and function of endogenous pulp progenitor cells in permanent molar teeth and found that the *Mx1* (myxovirus resistance 1) can be induced to selectively label endogenous pulp progenitor cells with *Cxcl12–*GFP expression. We further sought to define how *Mx1-*labeled *(*hereafter *Mx1*^+^) pulp progenitor cells respond to external stimuli and contribute to the replacement of odontoblasts and found that *Mx1*^+^ pulp progenitor cells are a main contributor to postnatal pulp cells and supply new odontoblast-like cells with reparative dentinogenesis under homeostatic and injury conditions.

## Materials and methods

### Animals

C57BL/6, *Mx1-*Cre^19^, *Rosa26*-loxP-stop-loxP-tdTomato (Rosa-Tom)^20^ and *LepR-*Cre^21^ mice were purchased from The Jackson Laboratory. *Osteocalcin–*GFP (*Ocn–*GFP, C57/BL6 background) mice were kindly provided by Drs. Ivo Kalajzic and Henry Kronenberg. *Cxcl12–*GFP mice were kindly provided by Dr. Takashi Nakasawa and *Scleraxis*–GFP (*Scx*–GFP) mice were generously shared by Dr. Brendan Lee. Genotyping of all Cre-transgenic mice and GFP fluorescent conjugated mice was performed by PCR (GenDEPOT) using primers for each sequence. Genotyping of the Rosa and GFP locus was performed according to The Jackson Laboratory’s protocols. For *Mx1*-Cre induction, the mice were injected twice intraperitoneally with 25 mg kg^−1^ of pIpC (Sigma) given at 2-day intervals. To exclude the effect of hematopoietic cells, the mice were lethally irradiated with 9.5 Gy 1 day before intravenous transplantation of 10^6^ whole bone marrow mononuclear cells from wild-type C57BL/6 mice (WT-BMT) followed by 6 weeks recovery. All mice were maintained in pathogen-free conditions, and all procedures were approved by Baylor College of Medicine’s Institutional Animal Care and Use Committee (IACUC).

### Histological analysis

The maxilla was dissected and fixed in 4% paraformaldehyde overnight at 4 °C, followed by decalcification in 10% EDTA for 7 days. Decalcified tissue was placed in a 10% sucrose solution overnight and moved to a 30% sucrose solution for storage. The samples were embedded in OCT Compound (Tissue-Tek, SAKURA) before sectioning. Cryosectioning was performed with a cryostat (CM3050S, Leica Biosystems) with the CryoJane tape transfer system (Leica Biosystems). For sections labeled with fluorescent reporters, the slides were stained with 4′,6-diamidino-2-phenylindole (DAPI) and visualized under the confocal microscope (A1R-s, Nikon) with filter cubes optimized for tdTomato, green fluorescent protein (GFP) and DAPI variants. Trichrome staining was performed using the trichrome stain kit (ab150686, Abcam), according to the manufacturer’s instructions and imaged by a bright field microscope (Ci-L, Nikon).

### Immunofluorescence

The frozen sections were air-dried and rehydrated with phosphate-buffered saline (PBS), blocked and permeabilized with 10% horse serum and 0.2% Triton X-100 in PBS at room temperature for 1 h. The sections were then probed with the following primary antibodies overnight at 4 °C: rabbit anti-Enpp6 (Proteintech, 12643-1-AP; 1:100), mouse anti-Crabp1 (Invitrogen, MA3-813; 1:200) and rabbit anti-Mx1 (Proteintech, 13750-1-AP; 1:20). Fluorescence-labeled secondary antibodies (1:1000) were incubated for 1 h under shielding from light at room temperature after washing with PBS.

### Mouse tooth injury

The mice were anesthetized with an intraperitoneal injection of an anesthetic combination of ketamine (56.3 mg kg^−1^), xylazine (2.9 mg kg^−1^) and acepromazine (0.6 mg kg^−1^) and prepared for the injury procedure on the maxillary first molars. All procedures for molar injury were performed on a three-dimensional-printed surgery bed^22^. A class I cavity was prepared with hand-operated micro drill bits (diameter, 0.20 mm) on the center of the mesial groove of the maxillary first molars^23^. According to the depth through which the drill bit penetrates, up to 0.1 mm is defined as a shallow cavity, and 0.1–0.2 mm is defined as a deep cavity. After reaching the appropriate depth, the cavity was washed with PBS to remove debris produced by cavity preparation and capped using light-cured composite resin (Flowable, Pentron) associated with a universal adhesive system (3 M).

### Flow cytometry analysis

To analyze the expression of cell surface markers, the mouse head was dissected, and all maxillary and mandibular first, second and third molars were extracted. After grinding the tooth in a mortar to separate the pulp tissue, the tissues were incubated with 5–10 ml of 0.1% collagenase and 10% fetal bovine serum in PBS at 37 °C for 1 h. The dissociated pulp cells were washed with PBS and filtered with a 40-mm strainer. Then the cell suspension was stained with mouse CD45-Pacific blue (clone: S18009F), CD31-Pacific blue (clone: 390), Ter119-BV605 (clone: TER-119), CD90.2-APC/Cy7 (clone: 30-H12), CD73-PE/Cy7 (clone: TY/11.8), CD29-APC (clone: HMb1-1), Sca-1-APC/Cy7 (clone: D7), CD200-APC (clone: OX-90) and CD146-PE/Cy7 (clone: ME-9F1). Flow cytometry experiments were performed using the LSRII and fluorescence-activated cell sorting (FACS) Aria cytometer (BD Biosciences). The data were analyzed with the FlowJo software (TreeStar) and represented as histograms, contour or dot plots of fluorescence intensity.

### Primary cell culture

The isolation process for mouse primary dental pulp cells follows the same protocol used for cell isolation in FACS analysis. The cells were seeded at a density of 1 × 10^3^ cells per well in 96-well plates and maintained in DMEM/10% fetal bovine serum medium at 37 °C with 5% CO_2_. The medium was changed every 2–3 days. The cells were subcultured when they reached 70–80% confluency. The plates were imaged under the confocal microscope (A1R-s, Nikon) with filter cubes optimized for tdTomato and GFP with DIC.

### Single-cell RNA sequencing analysis

#### Cell isolation and sequencing

Molars of P25, including both maxillary and mandibular M1, M2 and M3, were digested to obtain the single-cell transcriptomes. Briefly, the molars of three mice were digested in 4 mg ml^−1^ dispase and 2 mg ml^−1^ collagenase I on a thermomixer at 37 °C for 30 min to 1 h, depending on the stage of the sample, to release the cells from the tissue. For each sample, 10,000 cells were targeted for single-cell RNA sequencing, with the actual sequenced cells at 11,058 cells. Quality control, mapping and count table assembly of the library were performed using the CellRanger pipeline version 6.1.2.

#### Variable genes and dimensionality reduction

Raw read counts from the cells at each stage were analyzed using the Seurat 4.3 R package. Data from P3.5 and P7.5 molars in the literature^24^ were merged and integrated with the P25 dataset. The cells with low gene expression were filtered out following standard Seurat object generation. Cells with <300 genes per cell and with more than 25% mitochondrial read content were filtered out. For the merged datasets, the PrepSCTIntegration function was performed before identifying anchors with the function FindIntegrationAnchors. Seurat objects were returned by passing these anchors to the IntegrateData function. RunPCA and RunUMAP visualizations were used for downstream analysis and visualization. For analysis of the P25 dataset only, Sctransform was applied for normalization and cell cycle regression. RunPCA and RunUMAP were performed for dimensionality reduction and final visualization of the clustering.

#### Subcluster analysis

Heterogeneity within the dental mesenchymal populations was investigated through subcluster analysis. Published markers for the dental mesenchyme were used to screen and identify the different dental mesenchymal cell populations.

For the integrative analysis of samples at different stages, Seurat 4 was used to combine the single-cell data from five stages and perform integration analysis. The PrepSCTIntegration function was run before identifying anchors with the function FindIntegrationAnchors. Seurat objects were then returned by passing these anchors to the IntegrateData function. RunPCA and RunUMAP visualization were used for downstream analysis and visualization.

#### Monocle trajectory analysis

Monocle was used for pseudotime trajectory inference across the dental mesenchymal cells. Monocle inferred cluster and lineage relationships within a given cell type through the inputted cells. The estimation of the root node of the trajectory was based on the stem cell markers such as *Cxcl12* and *Pdgfra*.

### Statistical analysis

All measurements were made by a blinded examiner at three independent trials, and the average was recorded. All data were expressed as the mean ± s.e.m. For comparison between two independent groups, statistical differences were evaluated by unpaired two-tailed Student’s *t*-test. One-way analysis of variance, followed by Tukey’s post hoc test, was performed for multiple comparisons. A *P* value <0.05 was considered as statistical significance.

## Results

### Posterupted dental pulp cells switched to CP-like characteristics with Mx1 expression

Dental pulp cells are known to originate from neural crest cells^24,25^. However, adult pulp cells have a unique environment with less defined endogenous function and identity. In particular, how the population and molecular characteristics of pulp cells change before and after eruption of a molar tooth has not been studied.

**Fig. 1:**
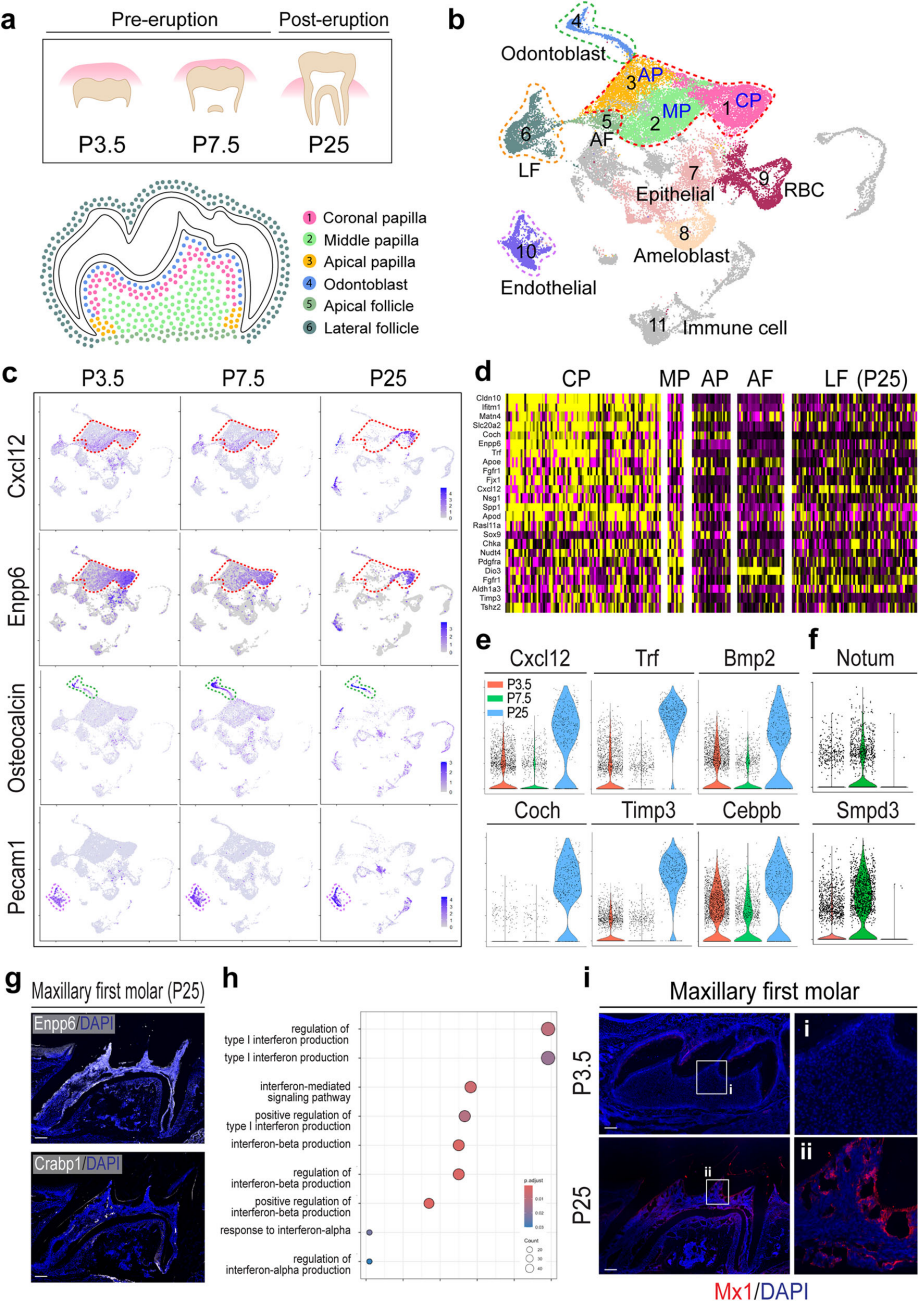
Cluster shifting in Single-cell analysis of pre- and posterupted molars relating to postnatal dental pulp progenitors. **a** A schematic diagram of molar eruption and single-cell sequencing from posterupted first molars (P25) compared with previously reported datasets from pre-eruption (P3.5) and eruption (P7.5) stages. The bottom is a schematic diagram identifying clusters present in the tooth germ in the first molar of the pre-eruptive stage. **b** An integrated UMAP visualization of 11 different color-coded clusters: cluster 1: CP, cluster 2: middle papilla (MP), cluster 3: apical papilla (AP) (all papilla clusters with blue dots), cluster 4: odontoblast (green dots), cluster 5: apical follicle (AF), cluster 6: lateral follicle (LF), cluster 7: epithelial, cluster 8: ameloblast, cluster 9: red blood cell (RBC), cluster 10: endothelial (purple dots) and cluster 11: immune cell. **c** UMAP-based transcriptional plots for *Cxcl12*, *Enpp6*, *Osteocalcin* and *Pecam1* at P3.5, P7.5 and P25. The dashed lines indicate the CP (red), odontoblast (green) and endothelial (purple) clusters at each time point. **d** Heat maps showing the top differentially expressed genes in the indicated clusters at P25 of five main clusters. **e**, **f** Violin plots for *Cxcl12*, *Trf*, *Coch*, and *Timp3* expression on (**e**) and *Notum* and *Smpd3* expression on (**f**) from the CP-like cluster at P3.5, P7.5 and P25. **g** A visulization of Enpp6 and Crabp1 immunofluorescence of maxilary first molar at P25. **h** Gene ontology over-representation test of P25 CP clusters shows the higher expression genes involved in type I interferon related pathways. **i** Anti-MX1 antibody staining (a target of type I interferon signaling, red) of P3.5 and P25 first molar sections. Blue, DAPI. The white boxes enlarged on the right sides. Scale bar, 200 μm.

To define molecular changes in pulp cells before and after tooth eruption, we performed single-cell RNA sequencing of the mouse maxillary and mandibular molar cells at the posteruption stage (P25, mature molars with fully mineralized enamel, mature roots and closed apex). We normalized and compared the results to previously reported datasets from the mouse molar cells at the pre-eruption stages (P3.5 and P7.5, immature molars with early root development)^24,26,27^ (Fig. 1a). A uniform manifold approximation and projection (UMAP) clustering analysis of integrated datasets showed that molar teeth comprised at least ten distinct clusters (Fig. 1b). Among these clusters, cluster 1 selectively expressed coronal papilla (CP) markers (*Enpp6*, *Fabp7* and *Fmod*), cluster 2 expressed middle papilla markers (*Nnat4*, *Rab3b* and *Vcan*) and cluster 3 expressed apical papilla markers (*Crabp1*, *Tac1* and *Omd*), annotating that cluster 1, 2 and 3 represent dental papilla cells that can develop into pulp cells and odontoblasts. An additional cell-type-specific marker analysis revealed that cluster 4 expressed odontoblast markers (*Ocn* and *Dmp*), and cluster 5 and 6 expressed dental follicle cell markers, while epithelial and ameloblast markers were enriched in cluster 7 and 8 and endothelial and immune cell markers were highly expressed in cluster 10 and 11, respectively (Fig. 1b and Supplementary Fig. 1a).

Next, we separately analyzed pre-eruption (P3.5 and P7.5) and posteruption (P25) datasets and examined pulp populational changes between pre- and posteruption of the permanent tooth. Notably, while P3.5 and P7.5 papillar cells comprised distinct apical papilla, middle papilla and CP-like clusters, a majority of P25 pulp cells exhibited a marked decrease in apical papilla and middle papilla markers but a proportional increase in CP markers (*Cxcl12* and *Enpp6*) (Fig. 1c). For the invariable controls, mature odontoblasts (cluster 4) and endothelial cells (cluster 10) did not change their numbers and marker expression throughout all time points, implicating that pulp progenitor cells in posterupted tooth obtain CP-like characteristics (Fig. 1c and Supplementary Fig. 1b).

Differential gene analyses of P25 pulp cells revealed that the CP-like cluster (cluster 1) in P25 pulp highly expresses stem cell markers (*Cxcl12*, *Enpp6* and *Ifitm1*) and positive regulators of odontoblastic differentiation (*Slc20a2*, *Cldn10* and *Timp3*) (Fig. 1d). Violin plot comparisons of coronal pulp cells revealed distinct gene expression patterns between the posteruption stage (P25) and pre-eruption stages (P3.5 and P7.5). At P25, there was increased expression of genes associated with stem cell maintenance and differentiation (*Bmp2* and *Cebpb*), extracellular matrix remodeling and tissue homeostasis (*Timp3* and *Coch*) and cell migration/metabolic support for regeneration (*Cxcl12* and *Trf*), along with decreased expression of genes related to mineralization and dentin maturation (Notum and Smpd3)^13,28–38^ (Fig. 1e, f). These findings were further validated by immunohistochemistry, which showed that P25 maxillary molar pulp cells predominantly express the CP marker Enpp6, while only a small subset express the apical papilla marker Crabp1 (Fig. 1g).

Notably, gene ontology enrichment test of P25 pulp cells showed that CP cluster has multiple activating gene sets associated with type I interferon and interferon-mediated signaling compared with other clusters (Fig. 1h and Supplementary Fig. 1c). Given *Mx1* is a well-established type I interferon stimulated gene^39^, we performed immunohistochemistry to examine the expression of the endogenous MX1 at pre-erupted (P3.5) and posterupted (P25) maxillary first molars. Consistantly, P25 pulp cells have increased expression of MX1 compared with P3.5 pulp cells (Fig. 1i). These results suggest that molar pulp progenitor cells undergo a unique population change after eruption and acquire the CP-like characteristics with higher expression of interferon-mediated signaling genes.

### Mx1^+^ progenitor cells contribute to the repopulation of pulp cells and odontoblasts

**Fig. 2:**
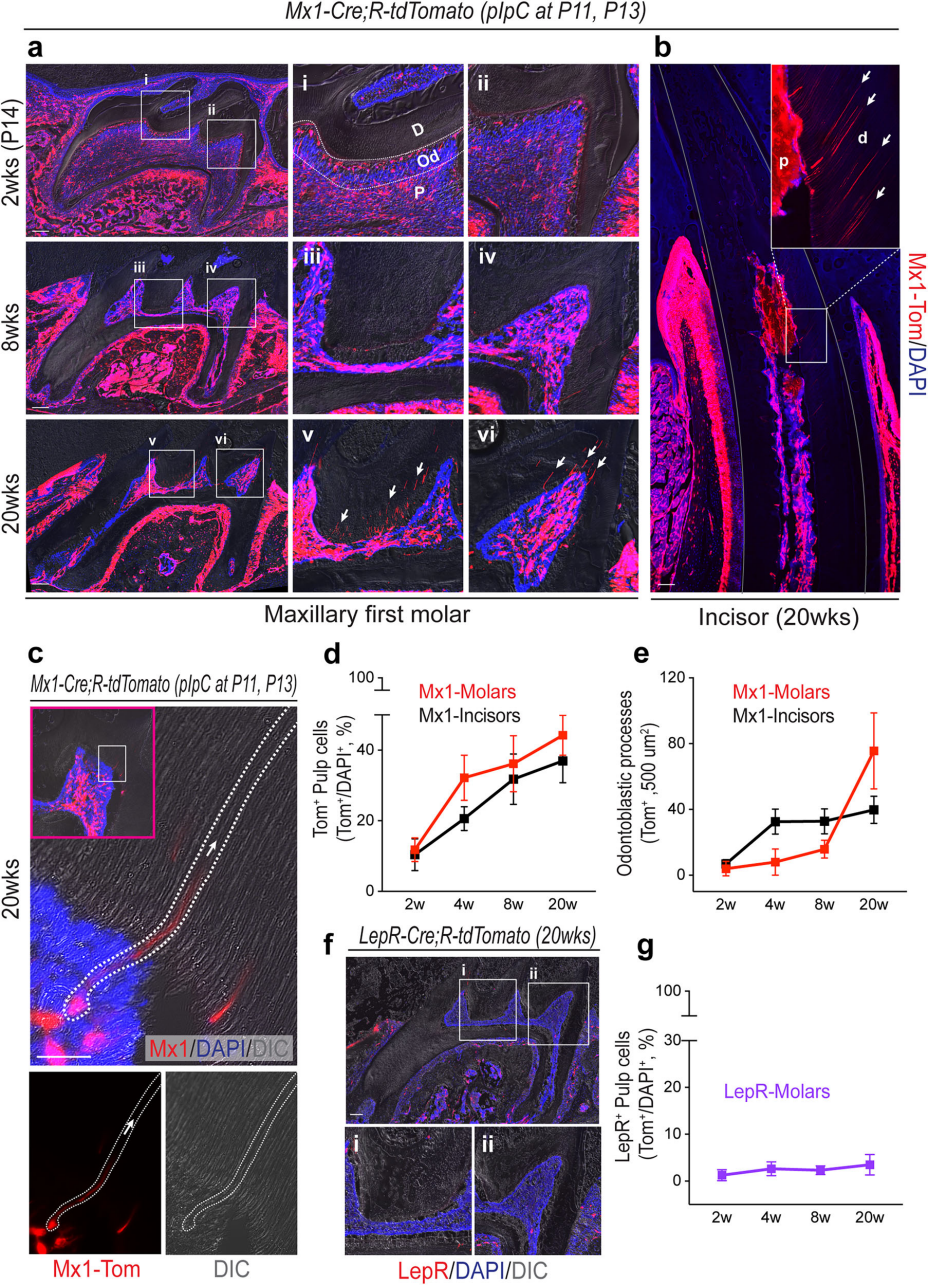
Postnatal contribution of *Mx1*^+^ cells on adult pulp cells and odontoblasts. **a** *Mx1*-Cre;*Rosa*-tdTomato reporter mice were pIpC-induced (at P11 and P13) and Tomato^+^ pulp cells in maxillary first molar (M1) were analyzed at the indicated time points (2 weeks (wks), 8 wks and 20 wks). The white boxes (i–vi) indicate enlarged images on the right side. *Mx1*^+^ odontoblastic processes (Tom^+^) on dentin area is indicated by white arrows (v–vi). D Dentin; Od Odontoblasts; P Pulp cells. **b** The expression patterns of *Mx1*^+^ cells in the mandibular incisor were determined at 20 wks of age. The white box is enlarged on the top right, and *Mx1*^+^ expressing odontoblast process within dentinal tubules (Tom^+^) are indicated by white arrows. **c** The high-magnification (100×) confocal image of *Mx1*^+^ odontoblast cell passing through pulp-dentin junction area. The pulp-dentin junction (red box) of maxillary first molar is enlarged and the path from the odontoblast cell body through the dentinal tubule is traced with a white dotted line. The white arrow indicate the direction of odontoblast process. The merged image is splited into the red (left bottom) and DIC (right bottom) channel. **d** The proportion of *Mx1*^+^ pulp cells in molars and incisors were calculated based on DAPI staining of whole pulpal cells from 2 wks through 20 wks. **e** The numbers of Tom^+^ cells in the odontoblast layer (Tom^+^ odontoblast cells) were counted by 500 µm^2^ of each molar and incisor sections (*n* = 5). **f** At 20 wks of age, *LepR*-Cre;*Rosa*-tdTomato reporter mice were analyzed to locate *LepR*^+^ cells in the dental pulp and dentin matrix. The white boxes are enlarged below the image (i, ii). **g** The percentages of *LepR*^+^ cells over DAPI-stained cells within the dental pulp (*n* = 5). The results are displayed as the mean ± s.e.m. Scale bar, 200 μm for **a**, **b** and **f**, 10 μm for **c**, respectively.

An important function of stem/progenitor cells is their long-term maintenance in vivo. The *Mx1*-Cre model has been known to inducibly label various adult stem cell populations, including hematopoietic, neural and skeletal stem cells (SSCs), with high expression of CXCL12 (refs. ^39,40^). Therefore, to assess whether *Mx1-*Cre model can label pulp progenitor cells in posterupted molars, we developed *Mx1*-Cre;*Rosa*-tdTomato (*Mx1-*Cre^+^;*Rosa26*-Tomato^f/w^) mice and induced with pIpC at P11 and P13 when the first molar in mice reaches maturity and starts to erupt. The postnatal effect of *Mx1*^+^ pulp cells from the posteruption stage was analyzed at 2, 4 and 16 weeks of age. At 2 weeks of age (1 day after pIpC labeling), only a small portion of the pulp cells (~5% ± 2%) in the maxillary first molar were *Mx1*-labeled (Tomato^+^) (Fig. 2a, 2 weeks, Tom^+^). However, the number and percentage of *Mx1*^+^ pulp cells continuously increased at 8 and 20 weeks of age (~20% at 8 weeks and ~80% at 20 weeks of age, respectively) (Fig. 2a, 8 weeks and 20 weeks, Tom^+^), while there were no detectable labeling of *Mx1*^+^ pulp cells without pIpC treatment (Supplementary Fig. 2a,b). Consistently, we found that over 40% of the pulp cells were *Mx1*^+^ cells in both apical and distal incisors (~40% at 20 weeks of age), suggesting that *Mx1-*Cre can inducibly label early postnatal pulp progenitor cells that are the major source of newly generated pulp cells (Fig. 2b, d). Notably, we observed that *Mx1*^+^ pulp cells developed new processes (tubule-like extensions) to reoccupy existing dentinal tubules in occlusal-side odontoblast layers at 20 weeks of age (Fig. 2a(v–vi), 20 weeks, white arrows, and Fig. 2c, e), which were undetactable at 4–8 weeks of age. In addition, similar *Mx1*^+^ odontoblastic processes (white arrows) were observed in the coronal region (near the tooth tip) but not in the radicular region (near the root) of mandibular incisors, at 20 weeks of age (Fig. 2b). It is possible that *Mx1*^+^ hematopoietic cells may contribute to the increase in *Mx1*^+^ pulp cells. To exclude this possibility, *Mx1*-Cre;*Rosa*-tdTomato mice were sublethally irradiated, and wild-type bone marrow cells were transplanted at 4 weeks of age. At 8 weeks of age, the number and percentage of *Mx1*^+^ pulp cells in first molars were not different compared with Mx1/Tomato mice without bone marrow transplantation (Supplementary Fig. 2c), further supporting that *Mx1* labels pulp progenitor cells that can contribute to new odontoblast-like cells on the occlusal-side of molars. By contrast, *Mx1*^+^ cells in the PDL contributed to the majority of PDL cells and alveolar osteoblasts, suggesting that *Mx1*^+^ follicle cells are similar to the previously identified PTHrP^+^ dental follicle progenitor cells^15,39^ (Supplementary Fig. 4a, b).

While dental pulp progenitor cells are proposed to be a promising cell source for cranial and jaw bone defects, whether endogenous pulp progenitor cells have skeletal progenitor characteristics is unknown. We previously found that *Mx1*^+^ SSCs highly overlap with *LepR*-Cre^+^ SSCs expressing *Cxcl12–*GFP^41^. Therefore, we next tested whether dental pulp progenitor cells express LEPR by using *LepR*-Cre^+^;*Rosa26*-Tomato^f/w^ mice. Interestingly, the first and second molars had little or no *LepR*-Cre^+^ cells (Tomato^+^) in the dental pulp and follicles at 2 weeks of age. Even at 20 weeks of age, we observed nearly no detectable *LepR*-Cre^+^ cells in the entire molar pulp, which indicates that *Mx1*^+^ pulp progenitor cells are distinct from *LepR*-Cre^+^ SSCs, with a unique microenvironment to regulate self-renewal and differentiation (Fig. 2f, g).

### Mx1 and Cxcl12 combination selectively labels endogenous pulp progenitor cells

**Fig. 3:**
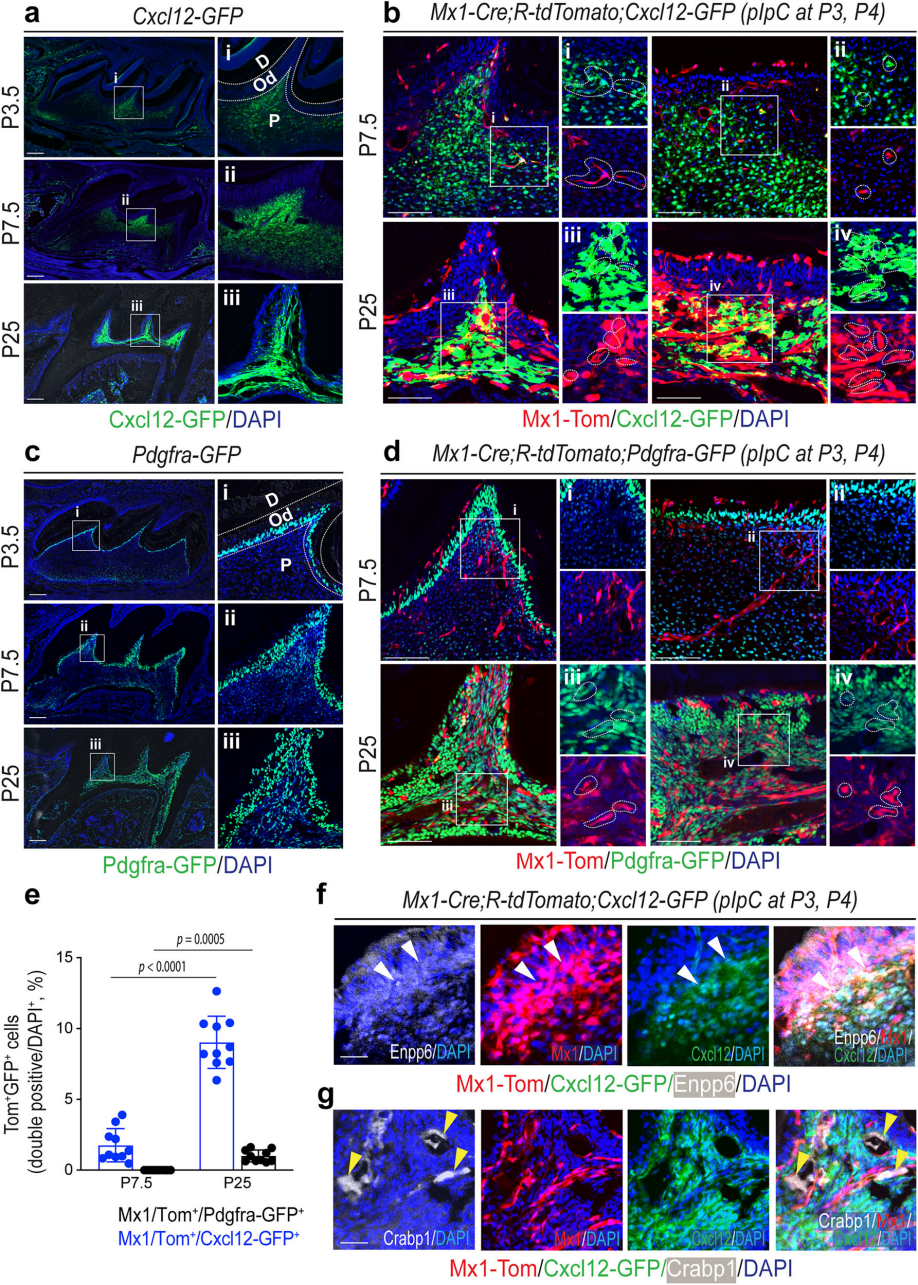
*Mx1-Cre* labels a subset of progenitor cells in dental pulp and odontoblast layer that express *Cxcl12*–GFP. **a** Representative fluorescence images of maxillary first molar of *Cxcl12*–GFP reporter mice at P3.5, P7.5 and P25. **b** *Mx1*-Cre;*Rosa*-tdTomato;*Cxcl12*–GFP reporter mice were pIpC-induced (P3 and P4). Representative fluorescence images of their maxillary first molars at P7.5 and P25 show the pulp horn (left) and pulp chamber roof (right) of molars. The white boxes to the right (i–iv) show enlarged images of *Mx1*^+^*Cxcl12*–GFP^+^ cells (i–iv, yellow dotted lines) with GFP (top) and Tomato (bottom) separation. **c** Representative fluorescence images of maxillary first molars of *Pdgfra*-H2B-GFP reporter mice at P3.5, P7.5 and P25. The white boxes (i–iii) are enlarged on the right side. **d** *Mx1*-Cre;*Rosa*-tdTomato;*Pdgfra*-H2B-GFP reporter mice were pIpC-induced (P3 and P4). Representative fluorescence images of their maxillary first molars at P7.5 and P25 show the pulp horn (left) and pulp chamber roof (right) of molars. Enlarged *Mx1*^+^*Pdgfra*–GFP^+^ cells in white boxes (i–iv, dot lines) are shown on the right side with GFP (top) and Tomato (bottom) separation (*n* = 10). **e** The graph shows the double-positive cells for each mouse line (*Mx1*^+^*Cxcl12*^+^ and *Mx1*^+^*Pdgfra*^+^) counted on the pulp and odontoblast layer. **f**, **g** Through immunohistochemistry, Enpp6 (**f**) and Crabp1 (**g**) antibodies were applied and visualized on *Mx1*-Cre;*Rosa*-tdTomato;*Cxcl12*–GFP reporter mice slide. The white (first), red (second) and green (third) channels were selectively activated with DAPI (blue). Right: the merged channels. The white wedges indicate the overlapped point with the staining of Enpp6. The yellow wedges indicate the staining of Crabp1 which is not overlapped with *Mx1* and *Cxcl12*. D Dentin; Od Odontoblasts; P Pulp for **a** and **c**, respectively. The results are displayed as the mean ± s.e.m. Scale bar, 200 μm for **a** and **c**, 100 μm for **b** and **d**, 50 μm for **f** and **g**, respectively.

The in vivo function of endogenous pulp progenitor cells remains elusive due to a lack of mouse models for pulp progenitor fate mapping. It has been know that, in the bone marrow, CXCL12 is highly expressed in reticular cells (CAR cells) and skeletal stem/progenitor cells^41^. Our single-cell analysis revealed CXCL12 as a key shifting molecule within the CP-like cluster, highlighting its potential as a progenitor cell marker (Fig. 1c–f). Therefore, we next tested whether endogenous pulp progenitor cells have a selective expression of CXCL12 in pre- and posterupted molars. By using the *Cxcl12–*GFP mouse model, we first examined which pulp cells express CXCL12 before and after eruption and found that a subset of central pulp cells expressed *Cxcl12–*GFP while mature odontoblasts were *Cxcl12–*GFP negative at P3.5. Interestingly, more pulp cells expressed *Cxcl12–*GFP at P7.5 and the majority of pulp cells highly expressed *Cxcl12*–GFP at P25, indicating that *Cxcl12–*GFP is a reliable marker for adult pulp cells but not specific to dental pulp progenitor cells (Fig. 3a). Therefore, to test whether the combination of *Mx1-*Cre and *Cxcl12–*GFP can label endogenous pulp progenitor cells, we developed trigenic *Mx1*-Cre;*Rosa*-tdTomato;Cxcl12–GFP reporter mice by crossing *Mx1-*Cre^+^;*Rosa26*-Tomato^f/f^ with *Cxcl12–*GFP^+^ mice. To identify the endogenous pulp progenitor population, we administrated pIpC at postnatal day 3 and 4 (pre-eruption stage) and examined the presence of double-positive cells (*Mx1*^+^*Cxcl12*^+^) and their repopulation capacity. When mice were pIpC-treated and the first molar was analyzed at P7.5, a small subset of *Cxcl12–*GFP^+^ cells (~2–3%) were *Mx1*^+^, and these double-positive cells exclusively resided in central pulp. Furthermore, the *Mx1*^+^*Cxcl12–*GFP^+^ cell population increased at P25 (~9–10%) while *Mx1* single-positive cells were enriched in the odontoblast layer (Fig. 3b, e).

Given PDGFRA was reported to be expressed in dental mesenchyme in tooth development^42^, we next tested the location of PDGFRA^+^ pulp cells by using *Pdgfra-*H2B-GFP mice and found that cells in odontoblast layers were mainly labeled by *Pdgfra-*H2B-GFP (Fig. 3c). To test whether *Mx1-*Cre labels these odontoblastic cells, we crossed *Mx1-*Cre^+^;*Rosa26*-Tomato^f/f^ mice with *Pdgfra-*H2B-GFP^+^ line. When Mx1/Tomato/Pdgfra*-*H2B-GFP mice were pIpC-induced at P3 and P4, there was no detectable *Mx1*^+^ cell contribution to *Pdgfra-*H2B-GFP^+^ in the pulp or odontoblast layer at P7.5, but the *Mx1*^+^*Pdgfra-*H2B-GFP^+^ cell numbers were increased at P25, indicating that early *Mx1*^+^ pulp progenitor cells at least minimally contribute to *Pdgfra-*H2B-GFP^+^ odontoblastic cells (Fig. 3d, e).

Further, immunohistochemistry of coronal and apical papilla markers showed that most *Mx1*^+^*Cxcl12–*GFP^+^ pulp cells in P25 molar highly overlap with Enpp6^+^ cells (a CP marker) but do not overlap with Crabp1 (an apical papilla marker), supporting that *Mx1*^+^*Cxcl12–*GFP^+^ cells are pulp progenitor cells (Fig. 3f, g). Interestingly, *Mx1*^+^ cells in dental follicles mainly contributed to PDL cells and without *Cxcl12–*GFP or *Pdgfra*-H2B-GFP expression, suggesting that the *Mx1* and *Cxcl12–*GFP combination can selectively label endogenous pulp progenitor cells while *Mx1*^+^ PDL progenitor cells are negative for *Cxcl12–*GFP (Supplementary Fig. 3a, b).

### Mx1^+^Cxcl12–GFP^+^ cells represent an enriched progenitor population with high stem cell marker expression

**Fig. 4:**
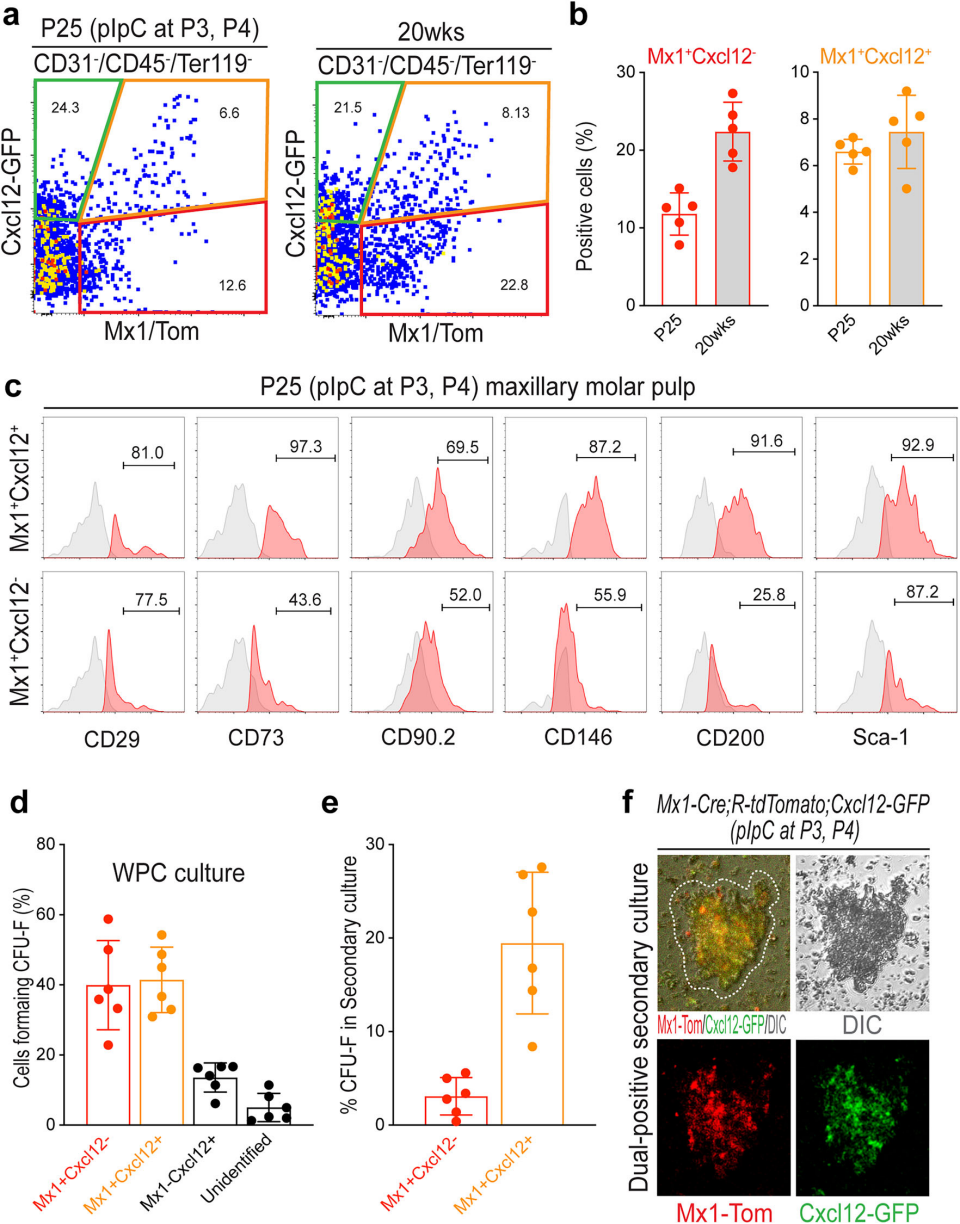
*Mx1*^+^*Cxcl12*^+^ progenitor cells express stem cell markers with high clonogenic potential. **a** FACS analysis was performed with the cells isolated from the maxillay and mandibular molars of *Mx1*-Cre;*Rosa*-tdTomato;Cxcl12–GFP reporter mice treated with pIpC (at P3 and P4) with the result of the P25 sample and 20-week sample. The cells were stained with CD200, CD146, Sca-1, CD90.2, CD73 and CD29 and fractioned with CD31-CD45-Ter119- selection. The cell population were divided with *Mx1*^+^*Cxcl12*^+^, *Mx1*^+^*Cxcl12*^−^, and *Mx1*^−^*Cxcl12*^+^ populations depending on the location in the Tomato–GFP scatter plot. **b** Each of the populations is expressed on the graph with different colors (legend on the top right), and each bar represents the proportion of single staining results. **c** The relative cell surface expressions of stem cell markers (CD29, CD73, CD90.2, CD146, CD200 and Sca-1) of *Mx1*^+^*Cxcl12*^+^ and *Mx1*^+^*Cxcl12*^−^ cells from P25 mice. *N* = 5 for each group. **d** The cells forming CFU-F were calculated as a percentage of whole dental pulp cells cultured. **e** *Mx1*^+^*Cxcl12*^+^ and *Mx1*^+^*Cxcl12*^−^ populations were selectively isolated and cultured in a secondary culture. Colony forming units were calculated for each of population (*n* = 6). **f** Top: one of the dual-positive cell colony found on a subculture (secondary culture) condition. Bottom: Tomato (left) and GFP (right) single color image of the same colony. The results are displayed as the mean ± s.e.m. Scale bar, 100 μm.

Previous studies have demonstrated that dental pulp progenitor cells possess many in vitro characteristics of mesenchymal stem cells^43–47^ and highly express stem cell markers including CD73, CD90 and CD200 (ref. ^48^). To test whether the *Mx1*^+^*Cxcl12–*GFP^+^ combination is a part of this fraction, FACS analysis of molar pulp cells from P25 and 20-week-old Mx1/Tomato/Cxcl12–GFP reporter mice revealed that the increased percentage of *Mx1*^+^*Cxcl12–*GFP^+^ cells within CD45^−^CD31^−^Ter119^−^ pulp cells (from 6.6% to 8.1%) and an almost twofold (from 12.6% to 22.8%) increase in the percentage of *Mx1*-single-positive cells at 20 weeks of age (Fig. 4a, b). When the *Mx1*^+^*Cxcl12–*GFP^+^ and *Mx1*^+^*Cxcl12*–GFP^−^ populations in maxillary molar were further analyzed for their stem cell marker expression (CD29, CD73, CD90.2, CD146, CD200 and Sca-1), the *Mx1*^+^*Cxcl12–*GFP^+^ population highly expressed stem cell markers (~80–90%) compared with *Mx1*^+^*Cxcl12*–GFP^−^ population (Fig. 4c). We also observed that *Mx1*^+^*Cxcl12–*GFP^+^ pulp cells retained long-term repopulation capacity in the CFU-F assay even after multiple passages (Fig. 4d–f). By contrast, *Mx1*^+^*Cxcl12*–GFP^−^ cells appeared to lose CFU-F ability after multiple passages, indicating that endogenous pulp progenitor cells are preferentially enriched in the *Mx1*^+^*Cxcl12–*GFP^+^ pulp cell population.

### Mx1^+^ progenitor cells contribute to new Ocn^+^ odontoblasts under homeostatic conditions

**Fig. 5:**
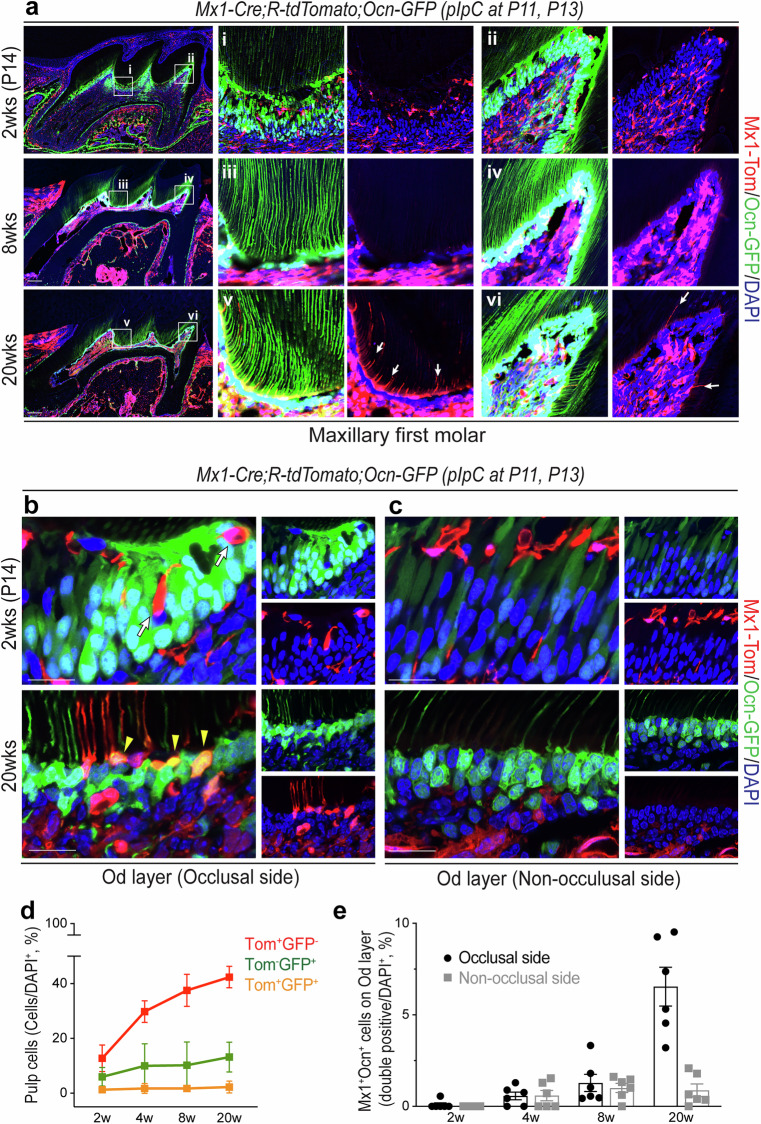
*Mx1*^+^ progenitor cells differentiate into mature *Ocn–*GFP^+^ odontoblasts. **a** pIpC-induced (at P11 and P13) *Mx1*-Cre;*Rosa*-tdTomato;*Ocn*–GFP reporter mice were analyzed with a fluorescence microscope; representative images of maxillary first molar at 2, 8 and 20 weeks (wks) of age with DAPI staining. Right: the white boxes were enlarged (i–vi) and split into Tom-DAPI channels. The white arrows show endogenous expression of *Mx1*^+^ cells (v–vi). **b**, **c** 100× Microscope images focused on odontoblast layers at 2 and 20 wks of age. Each of the images were split into GFP–DAPI (top) and Tom-DAPI (bottom) channels on the right side. Pulp chamber roof (occlusal side) of odontoblast layers (**b**) include *Mx1*^+^ originating odontoblast cells (white arrow, 2 wks) and their mature form (yellow wedge, 20 wks) with odontoblastic process from cell bodies. The odontoblast layers for lateral side of maxillary first molars (nonocclusal side) (**c**) have less *Mx1*^+^ originating cell intervention, as well as *Ocn*–GFP expression, on residual odontoblasts compared with the occlusal side. **d** The percentages of Tom^+^GFP^−^, Tom^−^GFP^+^ and Tom^+^GFP^+^ cells over DAPI staining within the dental pulp at different ages were calculated based on sections. **e** The numbers of Tom^+^GFP^−^, Tom^−^GFP^+^ and Tom^+^GFP^+^ cells within the odontoblast layer at different ages were counted based on sections (500 μm^2^, *n* = 5). The results are displayed as the mean ± s.e.m. Scale bar, 200 μm for **a** and **f**, 10 μm for **b** and **c**, respectively.

We next tested whether *Mx1*^+^ progenitor cells contribute to new odontoblast-like cells or whether *Mx1-*Cre can label mature odontoblasts that later develop odontoblastic processes. Given that mature odontoblasts were reported to express *Osteocalcin*–GFP (*Ocn–*GFP)^49^, we developed a trigenic *Mx1*-Cre^+^;*Rosa26*-Tomato^f/w^;*Osteocalcin–*GFP^+^ (*Mx1*-Cre;*Rosa*-tdTomato;*Ocn*–GFP) mouse model. In this model, *Mx1*^+^*Ocn*–GFP^−^ progenitor cells are Tomato single positive (Tom^+^GFP^−^), and when *Mx1*^+^ progenitor cells differentiate into odontoblasts, they become *Mx1*^+^*Ocn*–GFP^+^ (Tom^+^GFP^+^) odontoblasts^50^. Immunofluorescence images of the maxillary first molar of 2-week-old *Mx1*-Cre;*Rosa*-tdTomato;*Ocn*–GFP mice (1 day post pIpC injection) showed that nearly all *Mx1*^+^ progenitor cells were Tomato single positive and resided in the central pulp. There was no *Mx1* labeling of existing odontoblasts (Tom^−^GFP^+^), although a subset of *Mx1*^+^ cells was located in the predentin area, which was only observed in premature dentin (Fig. 5a, 2 weeks). However, at 8 weeks of age, we observed that *Mx1*^+^*Ocn*–GFP^−^ progenitor cells repopulated in the dental pulp and *Mx1*^+^*Ocn*–GFP^+^ (double-positive) cells appeared in odontoblast cell layers (Fig. 5a, 8 weeks). Notably, at 20 weeks of age, we found a marked increase in the number of *Mx1*^+^*Ocn*–GFP^−^ pulp progenitor cells and *Mx1*^+^*Ocn*^+^ odontoblasts with the extension of Tomato^+^ processes (Fig. 5a, 20 weeks, white arrow), suggesting that *Mx1*^+^*Ocn*–GFP^−^ pulp progenitor cells move to the odontoblast layer and differentiate into new *Ocn*^+^ odontoblasts (Fig. 5d). High-magnification images (100×) further defined undifferentiated *Mx1*^+^*Ocn*–GFP^−^ cells within the odontoblast layer at 2 weeks of age (Fig. 5b, 2 weeks, Tom^+^GFP^−^, arrows) and their *Ocn*–GFP^+^ odontoblast differentiation (Tom^+^GFP^+^) with distinct processes at 20 weeks of age (Fig. 5b, 20 weeks, yellow wedges). Interestingly, these new *Mx1*^+^*Ocn*–GFP^+^ odontoblasts were enriched in the loaded plane of the molar (Fig. 5b, e, occlusal side) but nearly undetectable in the unloaded plane of the molar (Fig. 5c, e, nonocclusal side), suggesting that exogenous mechanical stimuli are required for the activation of *Mx1*^+^*Ocn*–GFP^−^ pulp progenitor cells and their odontoblast differentiation over time in adult mice. By contrast, *Mx1*^+^ cells within the PDL only contributed to *Ocn*–GFP^+^ cells in the alveolar bone surface but not to *Ocn*^+^ cells in cementum layers of PDL at 20 weeks of age (Supplementary Fig. 4c, d), further supporting that *Mx1*^+^ pulp progenitor cells differ from endogenous PDL progenitors that can differentiate into cementoblasts^15^.

### Mx1^+^Ocn^−^ cells are durable progenitor cells in adult dental pulp

**Fig. 6:**
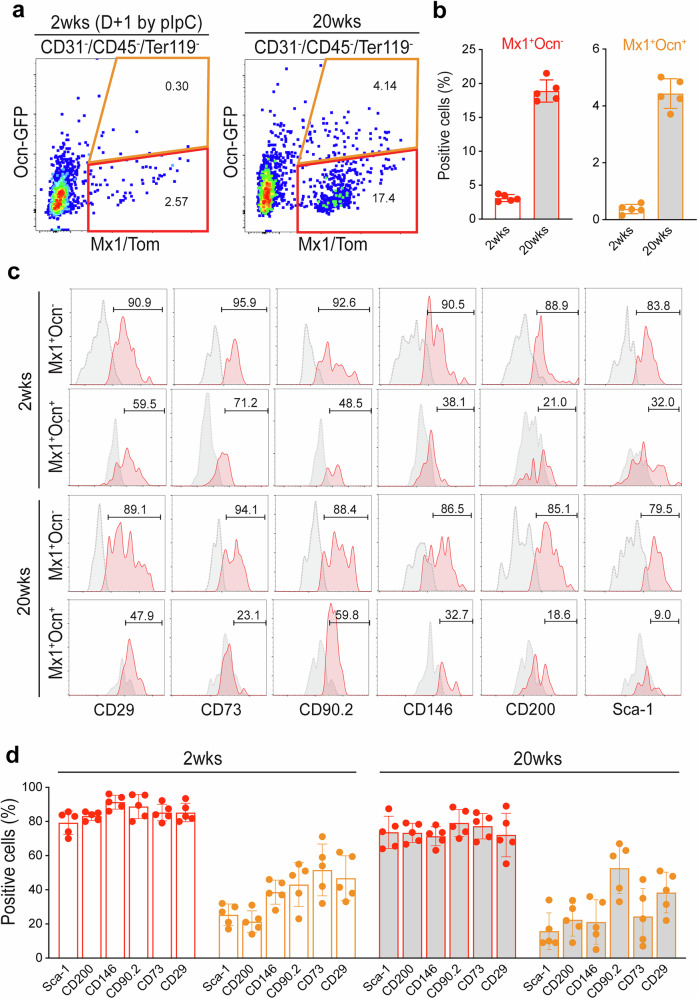
*Mx1*^*+*^*Ocn*^–^ progenitor cells preserve the expression of stem cell markers. **a** 2- and 20-week-old molar pulp cells of *Mx1*-Cre;*Rosa*-tdTomato;*Ocn*–GFP reporter mice were analyzed by FACS with CD31, CD45 and Ter119 negative fraction and segregated with Tomato (*x*-axis) and GFP (*y*-axis). The red boxes indicate the Tom^+^GFP^−^ population and the gray boxes the Tom^+^GFP^+^ population. **b** The collected repeatedly analyzed FACS data. The mean ± s.e.m. from three independent experiments with 3–5 mice per session is shown. **c** Relative cell surface expressions of stem cell markers (CD29, CD73, CD90.2, CD146, CD200, and Sca-1) of *Mx1*^+^*Ocn*^+^ and *Mx1*^+^*Ocn*^−^ cells from 2- and 20-week-old mice. *N* = 5 for each group. **d** The expression patterns of **c**.

It remains unkown whether endogenous pulp progenitor cells maintain their chracteristrics and physiological function in adult teeth. Therefore, we next examined whether *Mx1*^+^ pulp progenitor cells maintain progenitor characteristics and their contribution to mature odontoblasts at different ages. An FACS analysis of molar pulp cells from 2- and 20-week-old *Mx1*-Cre;*Rosa*-tdTomato;*Ocn*–GFP reporter mice revealed that only 2.57% (2.8 ± 0.6, *n* = 5) of CD45^−^CD31^−^Ter119^−^ pulp cells were *Mx1* positive at 2 weeks of age. However, this percentage increased almost sixfold (~17.4%) at 20 weeks of age (Fig. 6a, b, red box). Furthermore, the percentage of the *Mx1*^+^*Ocn*–GFP^+^ odontoblast-like population was increased by ~14-fold from 2 to 20 weeks of age (Fig. 6a, b, yellow box), which is consistent with our previous finding of an increased number of double-positive cells with age (Fig. 5a). Subsequent stem cell marker analysis revealed that most (~80–90%) *Mx1*^+^*Ocn*–GFP^−^ progenitor cells expressed stem cell immunophenotypic markers (CD29, CD73, CD90.2, CD146, CD200, and Sca-1), and this expression was durable even after 20 weeks of age (Fig. 6c, d), suggesting that *Mx1*^+^*Ocn*–GFP^−^ cells include durable pulp progenitor cells.

### Mx1^+^ progenitor cells differentiate into odontoblast-like cells to replace damaged odontoblasts at the injury site

**Fig. 7:**
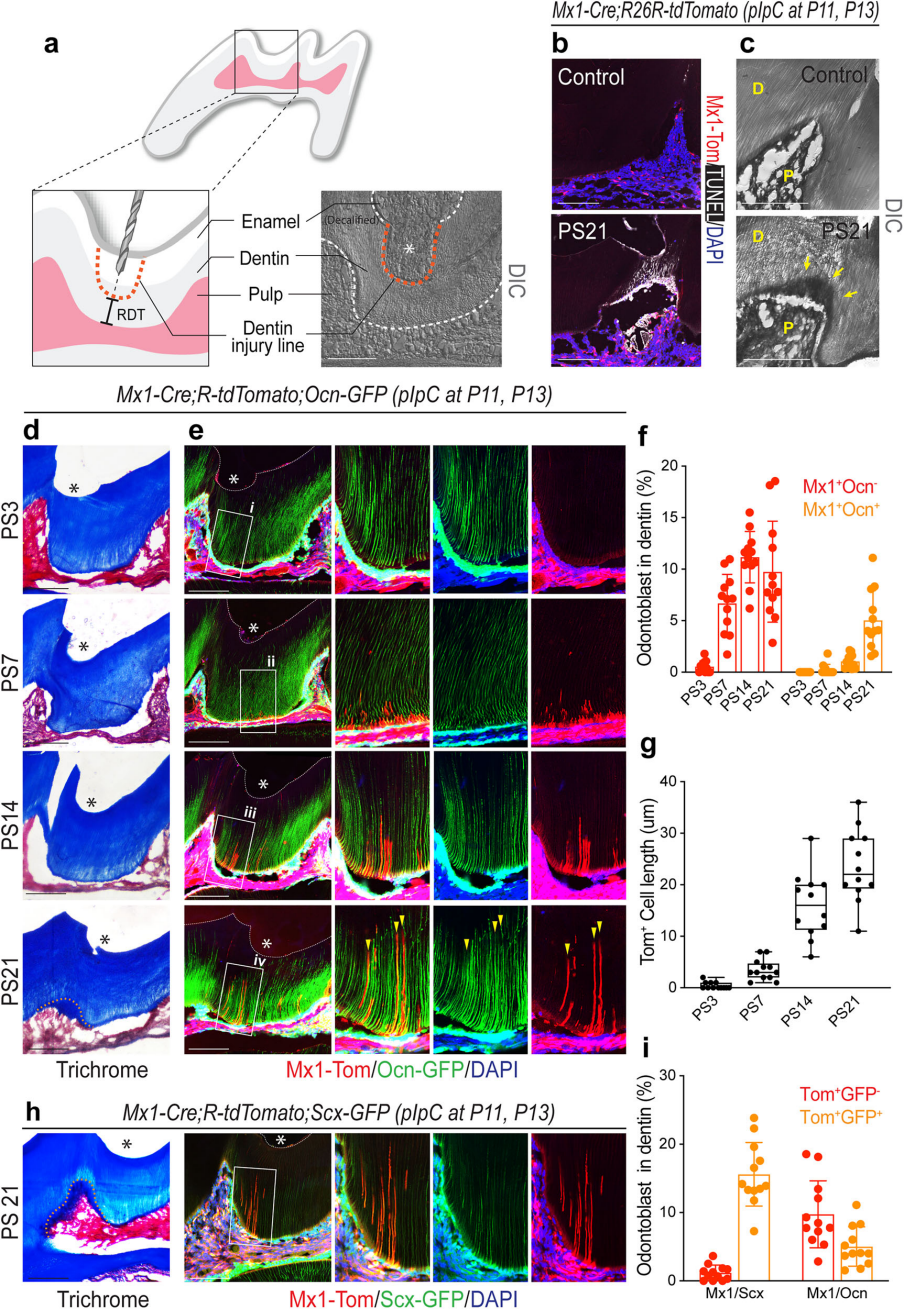
*Mx1*^+^ progenitor cells supply the majority of new *Ocn*^+^odontoblast-like cells in dentin injury. **a** A schematic diagram of a site-specific injury point on a maxillary first molar. The dentin injury lines are indicated (orange dashed line) with overall tooth structure of right after the injury. **b**, **c** When observed 21 days after performing the site-specific injury, clusters of dead cells (white staining) were found on the dentinal tubules within the injured dentin through the TUNEL assay (**b**), and an unorganized mineral structure (yellow arrows) was found through the DIC view (**c**). D Dentin; P Pulp. **d, e** A site-specific injury was formed on the maxillary first molar mesial groove to pIpC-induced (at P11 and P13) 4-week-old *Mx1*-Cre;*Rosa*-tdTomato;*Ocn*–GFP reporter mice with 100–200 μm RDT depth, and the responses were analyzed by section imaging from day 3 to 21 post surgery. Trichrome staining (**d**) shows the anatomical position of the pulp and dentin as well as the injury point (*) located in the first molar mesial groove for each time point. The yellow dashed lines of trichrome staining (dark blue) indicate the formation of reparative osteodentin underneath the injury sites (PS21). Fluorescence section images with DAPI staining (**e**) matched with the same slide in **d**. For each time point, the injury points (*) are marked. Right: the white boxes are enlarged (i–iv). The enlarged image was split again into different channels with Tom-GFP-DAPI (left), GFP–DAPI (middle) and Tom-DAPI (right). The yellow wedges mark the points where elongated odontoblast process of *Mx1*^+^ originating cells with mature form (iv). **f** The total number of Tom^+^ (GFP^+^ or GFP^−^) cells in the dentinal tubule structure on each 100 μm^2^ section (*n* = 10). **g** The average length of Tom^+^ cells in the dentinal tubules on each 100 μm^2^ section (*n* = 10). **h** A site-specific injury was made on the maxillary first molar mesial groove to pIpC-induced (at P11 and P13) 4-week-old *Mx1*-Cre;*Rosa*-tdTomato;*Scx–*GFP reporter mice. The *Mx1*^+^ cell response and its overlap with *Scx–*GFP are indicated with trichrome staining (left) and fluorescence imaging (right) on PS21. The yellow dashed lines on trichrome staining indicate a reparative dentin line. The white box on fluorescence image is enlarged and split into different channels with Tom-GFP-DAPI (left), GFP–DAPI (middle) and Tom-DAPI (right). **i** The comparison expression and cell numbers of Tom^+^GFP^−^ or Tom^+^GFP^+^ cells between *Mx1*^+^ with or without *Scx*–GFP or *Ocn*–GFP on PS21. Each of the counting performed on 100 μm^2^ sections (*n* = 10). Scale bar, 100 μm.

Replacement of dying odontoblasts with reparative dentin formation is a critical process to protect teeth after injury. Therefore, to examine whether *Mx1*^+^ pulp progenitor cells contribute to the replacement of damaged odontoblasts and dentin repair in vivo, we generated tooth dentin injuries at the mesial groove on the maxillary first molar of 4-week-old *Mx1*-Cre;*Rosa*-tdTomato;*Ocn*–GFP mice. We used a hand-operated micro drill bit and generated uniform molar injury without exposing the pulp cavity. We measured residual dentin thickness (RDT) at the injured site and considered 100–200 m RDT as a moderate injury and all of the injuries were performed under the this condition (Fig. 7a–c). Histological analysis of molars at day 3 post injury revealed an increase in *Mx1*^+^ progenitor cells in the pulp chamber of severe defect sites without odontoblast differentiation (*Ocn–*GFP^−^) (Fig. 7d–g, PS3). Interestingly, at days 7 and 14 post injury, *Mx1*^+^ progenitor cells started to differentiate into new odontoblast-like cells with development of early processes in the dentin matrix boundaries at the injury sites (Fig. 7d–g, PS7–PS14). On day 21 post injury, *Mx1*^+^ odontoblast-like cells became fully polarized with the extension of their processes to replace demaged odontoblasts and a new subset of *Mx1*^+^ odontoblasts expressing *Ocn–*GFP (Tom^+^GFP^+^) (Fig. 7d–g, PS21, yellow wedges). This expression was further clarified by high-magnification images (Supplementary Fig. 5a). However, there were no detectable *Mx1*^+^ odontoblast-like cells in uninjured controls (Supplementary Fig. 5e). The Ki67 staining further confirmed active cell proliferation in the odontoblast layer at the injured surface (Supplementary Fig. 5c). Moreover, trichrome staining revealed the formation of a reparative dentin-like structure at the injury site, characterized by *Mx1*^+^ odontoblasts on day 21 (Fig. 7d, PS21, trichrome, dotted line), similar to reparative osteodentin mineralization described in previous studies^51,52^ (Supplementary Fig. 5d, yellow arrows).

While mature odontoblasts express many osteoblastic genes including Col1, ALP, DMP and Ocn, they are functionally and morphologically different from bone osteoblasts^53–55^. Interestingly, when we screened for odontoblast gene expression (Fig. 1c), we found that mature odontoblasts expressed *Scleraxis*, a known tendon/ligament marker. Therefore, we performed an injury experiment using *Mx1*-Cre;*Rosa*-tdTomato;*Scx–*GFP reporter mice and found that *Mx1*^+^ progenitor cells became *Mx1*^+^*Scx–*GFP^+^ odontoblast-like cells with the extension of odontoblastic processes (white box) and reparative dentin formation at the site of injury (Fig. 7h, i and Supplementary Fig. 5b). Taken together, these results suggest that *Mx1*^+^ pulp progenitor cells are a main source of fully functional new odontoblast-like cells reoccupying existing dentinal tubules and dentin formation following molar injury. As a control, when *Mx1-*Cre^+^;*Rosa26*-Tomato^f/w^ mice were pIpC-treated at P25 and P27 and analyzed *Mx1* labeling at P28, we found that the labeling of *Mx1*^+^ pulp cells was similar to the mice with pIpC treatment at P11 and P13 (Supplementary Fig. 2d). Further, none of the mice showed *Mx1*^+^ odontoblasts with processes, confirming the contribution of *Mx1*^+^ cells to new odontoblast-like cells in injury.

### Mx1^+^Cxcl12–GFP^+^ pulp progenitor cells serve as the major source of new odontoblast in adult molars

**Fig. 8:**
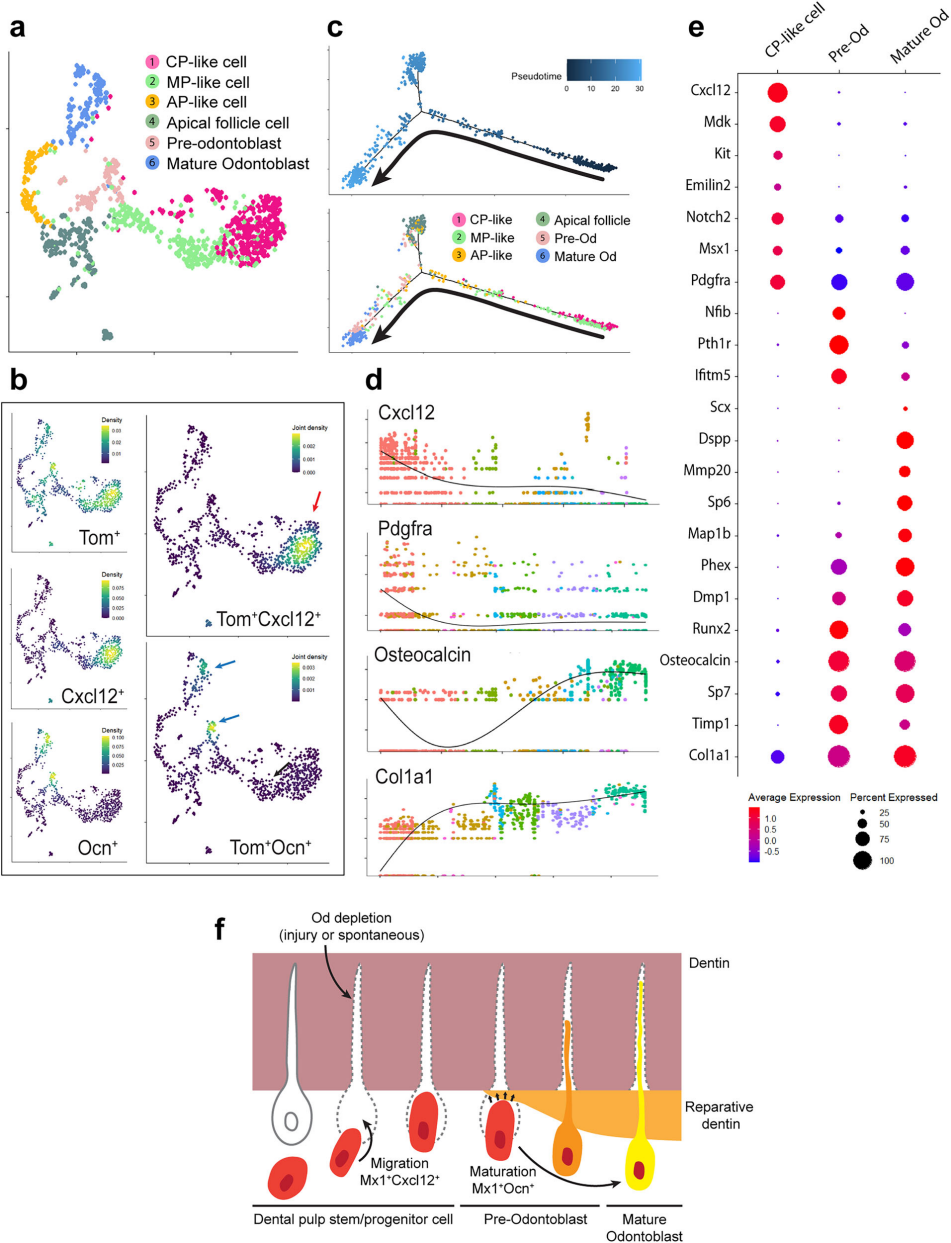
*Mx1*^+^ pulp progenitor cells are CP-like *Cxcl12*^+^ cells driving to mature odontoblast cells. **a** Single-cell analysis of *Mx1*-Cre;*Rosa*-tdTomato;*Ocn*–GFP first molar pulp at P25 and the UMAP visualization of six different color-coded clusters of odontoblast related groups. Cluster 1: CP, cluster 2: middle papilla, cluster 3: apical papilla, cluster 4: apical follicle, cluster 5: preodontoblast, cluster 6: odontoblast. **b** UMAP-based transcriptional plots for Tomato (Tom^+^), *Cxcl12*^+^, *Ocn*^+^ expression and joint plots for Tom^+^*Cxcl12*^+^ and Tom^+^*Ocn*^+^ expression. The black arrows on joint plots indicate points of the greatest coexpression. **c** The monocle pseudotime analysis of six clusters (top) and the computational model of cell fate decision result with the differentiation trajectory analysis of the different clusters at P25 (bottom). The black arrow is the follow-up of the trajectory of the main cluster according to pseudotime analysis. **d** Monocle pseudotime trajectory expression pattern of representative differentiation genes. The *x* axis represents pseudotime, and the *y* axis represents relative gene expression levels. **e** The gene expression screening for CP, preodontoblast or mature odontoblast clusters. The analyzed genes were previously identified as stem/progenitor markers for dental pulp (*Cxcl12* to *Pdgfra*) and odontoblast related markers (Nfib to Col1a1). **f** A schematic diagram of migration of *Mx1*^+^*Cxcl12*^+^ cells (dental pulp stem/progenitor cells) and their maturation to *Mx1*^+^*Ocn*^+^ cells (mature odontoblasts).

While *Mx1*^+^ pulp progenitor cells differentiate into odontoblast-like cells in adult molars, the origin of this cell population is not clear. Therefore, we performed single-cell sequencing of pulp cells from *Mx1*-Cre;*Rosa*-tdTomato;*Ocn*–GFP first molars at P25 (posteruption stage). A UMAP clustering analysis of the single-cell sequencing data showed six distinct clusters (Fig. 8a). Among these clusters, pulp cell markers and subsequent Tomato (*Mx1*^+^) and GFP (*Ocn*^+^) expression analysis revealed cluster 1 as CP-like cell, cluster 2 as middle papilla-like cell, cluster 3 as apical papilla-like cell, cluster 4 as apical follicle cell, cluster 5 as preodontoblast and cluster 6 as mature odontoblast, respectively (Fig. 8a and Supplementary Fig. 6a). Notably, Cxcl12 expression highly overlapped with Tomato (*Mx1*^+^) expression, and nearly all *Mx1*^+^*Cxcl12*^+^ cells were enriched in cluster 1. This suggests that cluster 1 represents an early pulp stem/progenitor population, which is clearly distinct from the *Ocn*^+^ or *Mx1*^+^*Ocn*^+^ cell population, a mature cell type (Fig. 8b, *Tom*^+^*Cxcl12*^+^ cluster, red arrow). Trajectory analysis of the entire cell population revealed that the CP-like cluster serves as a major source of progenitor cells for mature odontoblasts (Fig. 8c). Supporting this, monocle analysis demonstrated a gradual decline in progenitor-associated markers such as *Cxcl12* and *Pdgfra*, alongside an upregulation of odontoblast markers including *Osteocalcin* and *Col1a1* (Fig. 8d). Differential gene expression analysis and heat map analysis among CP-like cells, preodontoblasts and mature odontoblasts further confirmed their differentiation trajectory^56^ (Fig. 8e and Supplementary Fig. 6c). Gene ontology (GO) analysis illustrated the distinct biological roles of CP-like and odontoblast clusters, particularly in mineralization-related processes (Supplementary Fig. 6b). We also observed the presence of distinct *Mx1*^+^*Ocn*–GFP^+^ odontoblast clusters, further supporting the differentiation of CP-like cells into new *Ocn*–GFP^+^ odontoblasts in posterupted permanent molars (Fig. 8b, *Tom*^+^*Ocn*^+^ cluster, blue arrow). Taken together, these results suggest that *Mx1* can mark endogenous pulp progenitor cells, which can differentiate into mature odontoblast-like cells during adult molar homeostasis and injury. These new odontoblast-like cells replace dying odontoblasts, undergo reparative dentinogenesis and deposit secondary dentin on the bottom of primary dentin (Fig. 8f).

## Discussion

Our findings revealed that at least a portion of adult odontoblasts in permanent teeth are replaced by newly developed odontoblast-like cells and that endogenous pulp progenitor cells maintain the regeneration of pulp cells and new odontoblast-like cells. Our study also demonstrated a transition of molar pulp cells, from middle and apical papilla cells in early development to CP-like cells with *Cxcl12* expression in the post-eruption stage. Notably, a subset of *Cxcl12–*GFP^+^ cells are adult pulp progenitor cells that can be labeled by *Mx1*, and these *Mx1*^+^*Cxcl12–*GFP^+^ pulp progenitor cells reside in the mid pulp at the early tooth eruption stage, expand and contribute to the majority of new pulp cells and odontoblast-like cells, forming reparative dentin after injury.

The presence of dental pulp stem/progenitor cells in adult teeth has long been suggested. Genetic lineage tracing studies with mouse incisor models showed that several distinct mesenchymal populations, including nerve-associated glial cells, periarterial *Gli1*^+^ cells and *NG2*^+^ pericytes^12,57,58^, contribute to reparative dentinogenesis. Recently, *αSMA*^+^ (α smooth muscle actin) perivascular cells were reported to contribute to reparative dentinogenesis in adult mouse molars^11^. However, other studies suggested that odontoblasts are postmitotic cells with certain neuronal features that are normally not replaced during the life of an organism^59–61^. In fact, permanent teeth undergo substantial environmental changes before and after eruption. It is not clear how tooth environmental changes affect the molecular and cellular function of pulp progenitor cells. Further, whether endogenous pulp progenitor cells are long-term repopulating cells to supply new odontoblasts to replace dying odontoblasts under stress conditions remains unknown. Our study showed that *Mx1* selectively labels pulp progenitor cells present in the pulp core but not mature odontoblasts at 2 weeks of age. However, at 20 weeks of age, a marked increase in *Mx1*^+^*Ocn*–GFP^+^ odontoblasts in the dentin matrix in mouse molars was observed (Fig. 5a). The development and extension of *Mx1*^+^*Ocn*–GFP^+^ odontoblastic processes later in life further supported *Mx1*^+^ pulp progenitor cells directly differentiate into *Ocn*–GFP^+^ odontoblasts. In addition, our data showed endogenous *Mx1*^+^ pulp progenitor cells remain relatively similar from 4 to 20 weeks of age, even in the constantly growing incisors, indicating that *Mx1*^+^ pulp progenitor cells include a population of long-term repopulating cells. Our data also showed a mild-but-continuous attrition during mastication or aging (20-week-old mouse) is one of the main causes of mature odontoblast depletion; these cells are continuously replaced by new odontoblast-like cells differentiated from *Mx1*^+^ progenitor cells rather than through odontoblast proliferation. Single-cell analysis of *Mx1*^+^ pulp cells further supports this finding. While *Mx1*^−^ apical papilla-like and middle papilla-like pulp cells may contribute to some odontoblast differentiation, our data suggest *Mx1*^+^ progenitor cells are the major source of new odontoblasts in adult molars, and their contribution to mature odontogenesis is significantly greater than other cell clusters (Fig. 8c).

While *Mx1* expression has been observed in neural crest-derived tissues, its role in dental papilla lineage specification remains unclear. Recent single-cell studies highlight dynamic neural crest lineage diversification during tooth morphogenesis^24^, suggesting that early neural crest-derived populations may contribute to postnatal pulp progenitors. To confirm the linage of *Mx1*^+^ cells, future work should combine lineage tracing, such as *Wnt1*-Cre for neural crest, with markers specific to mesenchymal (for example, *Prx1* (ref. ^62^)) and ectomesenchymal neural crest cells (for example, *Sox10* (ref. ^63^) and *Pax7* (ref. ^64^)). Moreover, future studies will be required to define whether *Mx1*^+^ pulp progenitor cells are descendent of neural crest origin, mesenchymal cells or derived from peripheral nerve-associated glia cells in vivo^12^.

Dental pulp stem/progenitor cells and SSCs share many osteogenic characteristics and marker expression such as *osterix* (*Osx*), *osteocalcin* (*OCN*), *osteopontin* (*OPN*), *collagen type 1* (*Col I*) and *dentin matrix acid phosphoprotein 1* (*DMP1*), suggesting that pulp progenitor cells can be used as a transplantable stem/progenitor cell source to treat alveolar bone defects^53–55^. However, pulp progenitor cells and odontoblasts are highly polarized cells with unique features such as odontoblastic process formation, and how they are molecularly and functionally different from osteoblasts has not been elucidated. Our study revealed for the first time that odontoblasts show unique expression of tenogenic marker *Scx–*GFP, although their GFP signal is weaker than those of tendons and PDLs. We also found that *LepR*-Cre, a well-known bone marrow stromal cell marker, does not label pulp progenitor cells in mouse molars, indicating that *Mx1*^+^ pulp progenitor cells have unique molecular characteristics and can differentiate into *Scx–*GFP^+^ and *Ocn–*GFP^+^ odontoblast-like cells. It remains to be elucidated whether *Scx–*GFP can mark all *Ocn–*GFP^+^ mature odontoblasts or *Ocn–*GFP^−^ preodontoblasts.

Our study also provides further insight into the natural healing mechanism in regenerative and reparative dentin formation and provides evidence of the active role of endogenous pulp progenitor cells in injury response. We used a first molar injury mouse model, generated with a hand-operated micro drill that can inflict physiological and homogenous injuries without heat generation and pulp exposure^11,65^. Interestingly, when the first molar has severe injuries characterized by a remaining dentin thickness (RDT) of 100–200 μm, the *Mx1*^+^ progenitor cells migrate into the dentinal tubules (red wedges) with *Ocn–*GFP and *Scx–*GFP expression observed by day 28 post injury (Fig. 7), suggesting that *Mx1*^+^ pulp progenitor cells rapidly respond to outer tooth injuries and contribute to new odontoblast-like cells in the replacement of injured odontoblasts.

Taken together, our study supports the notion that there are both intrinsic and extrinsic cues that regulate the functions of adult pulp stem/progenitor cell subsets, and these progenitor cells might have an essential role in maintaining a reparative new odontoblast population with a distinct regulatory mechanism under stress conditions. Once identified, these regulatory mechanisms could be utilized to improve natural pulp healing and develop a novel therapeutic approach replacing the material base treatment of modern clinical dentistry.

### Limitations of the study

While this study revealed the exisitance of adult pulp progenitor cells with *Mx1* labeling, it is possible that the *Mx1*-labeled cells are heterogeneous and therefore additional stem cell markers will be further investigated in the subsequent work to expand the findings. In addition, the mechanisms of adult dental pulp progenitor maintenance and their odontogenic diffrentiaiton still remain unknown. Follow-up experiments should be conducted to address the above concerns and provide further evidence for the clinical application of dental pulp progenitor cells in permanent tooth health.

## Supplementary information

Supplementary Information
