# Supplementary material for: Mx1-labeled pulp progenitor cells are the main contributors of odontoblast and dentin regeneration in murine molars
Source: Exp Mol Med. 2025 Aug 13;57(8):1802–17. doi: 10.1038/s12276-025-01511-3 (PMC12411636; doi:10.1038/s12276-025-01511-3)
Supplement: Supplementary file 1 — Supplementary Information [file 12276_2025_1511_MOESM1_ESM.pdf]

SUPPLEMENTARY FIGURE 1

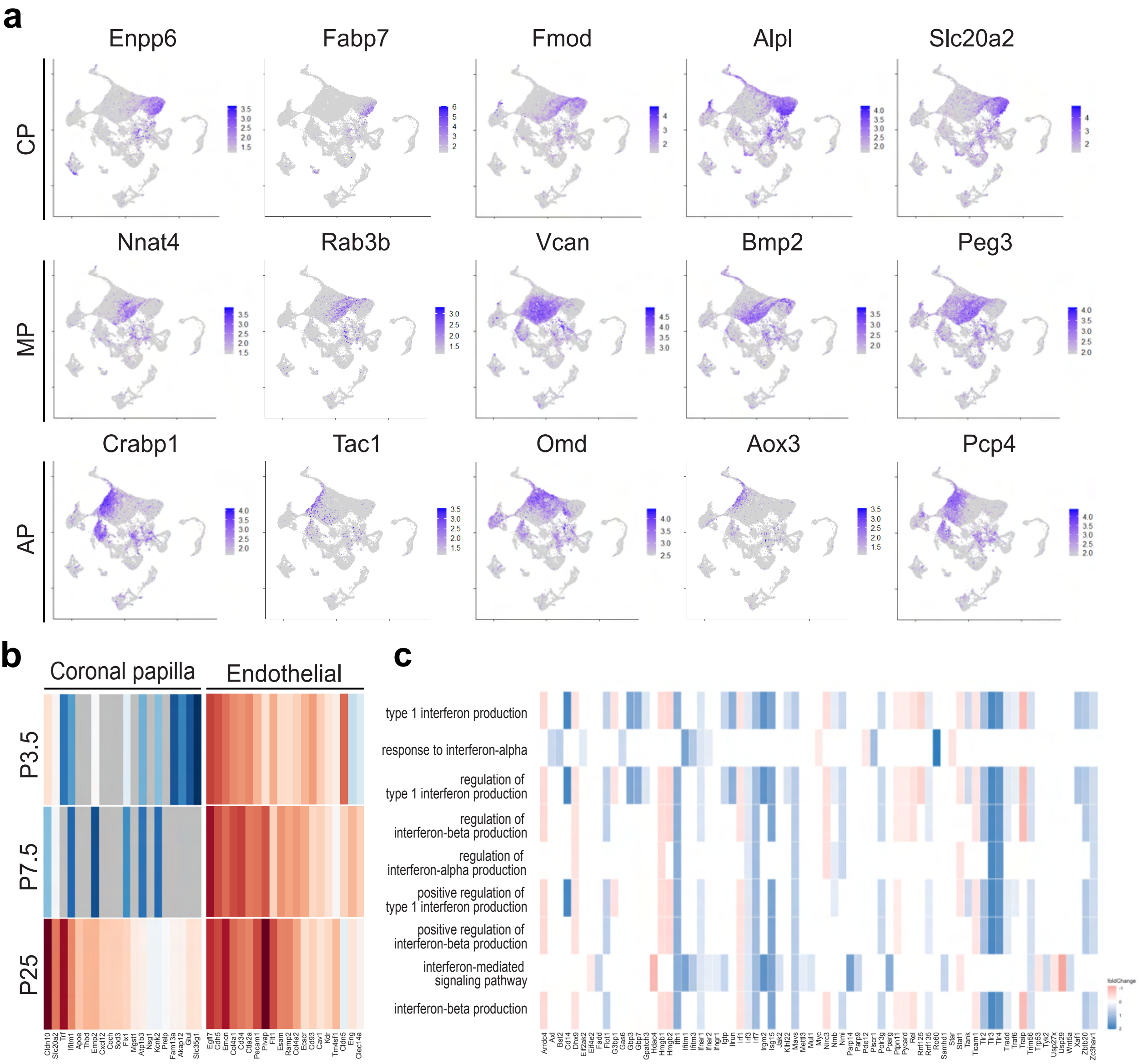

Supplementary Fig. 1: (a) UMAP plots of relevant markers for each dental papilla clusters (Apical, Middle, Coronal papilla). (b) Heatmap presentation of top 20 genes with induced expression in the coronal papilla cluster at P25 and genes without expression changes in the endothelial cluster at the indicated time points. (c) Heatmap of representative genes and fold changes of gene ontology of biological terms involved in type I interferon and interferon-alpha/beta related pathways in coronal papilla cluster.

SUPPLEMENTARY FIGURE 2

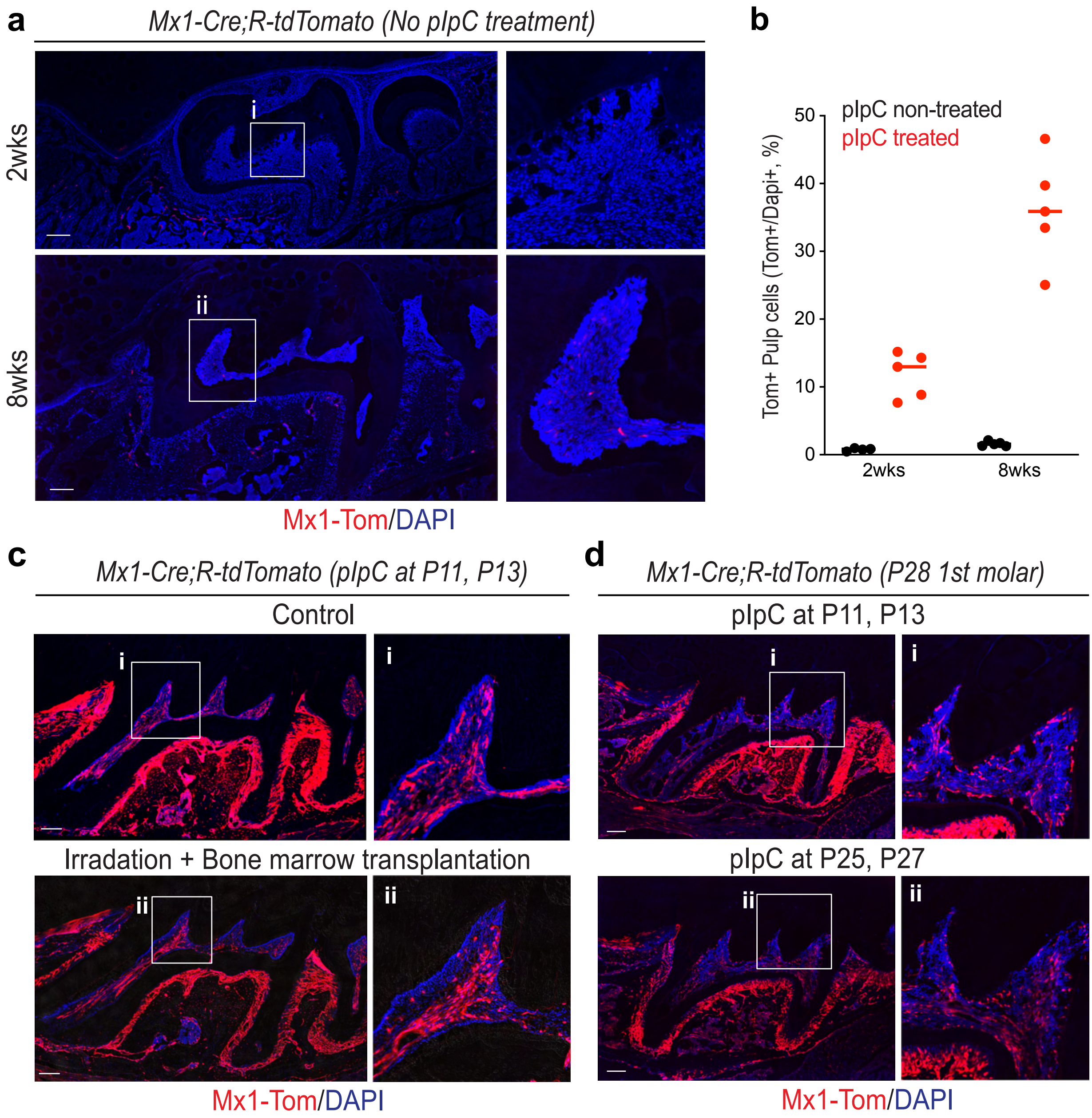

Supplementary Fig. 2: (a) The whole maxillary first molar tooth section of Mx1-Cre;Rosa-td-Tomato reporter mice at 2wks and 8wks, without pIpC induction. The enlarged images are located on the right side (i, ii). (b) The number of Tomato+ cells on (a) were counted and calculated by dapi (%). The data was compared with pIpC treated slides (P11,P13). (c) The whole maxillary first molar section of pIpC induced (P11, P13) 8wks old Mx1-Cre;Rosa-tdTomato reporter mice with irradiation followed by bone marrow transplantation. Mx1+ expressions were indicated on enlarged pulp (i,ii). (d) Control experiment with pIpC treatment on different time point to comparison between P28 molar with pIpC treatment on P11, P13 (Upper), and on P25, P27 (Lower). Samples were sacrificed on P28. Dental pulp (i,ii) were enlarged on the right side. Scale bar: 200 μm.

SUPPLEMENTARY FIGURE 3

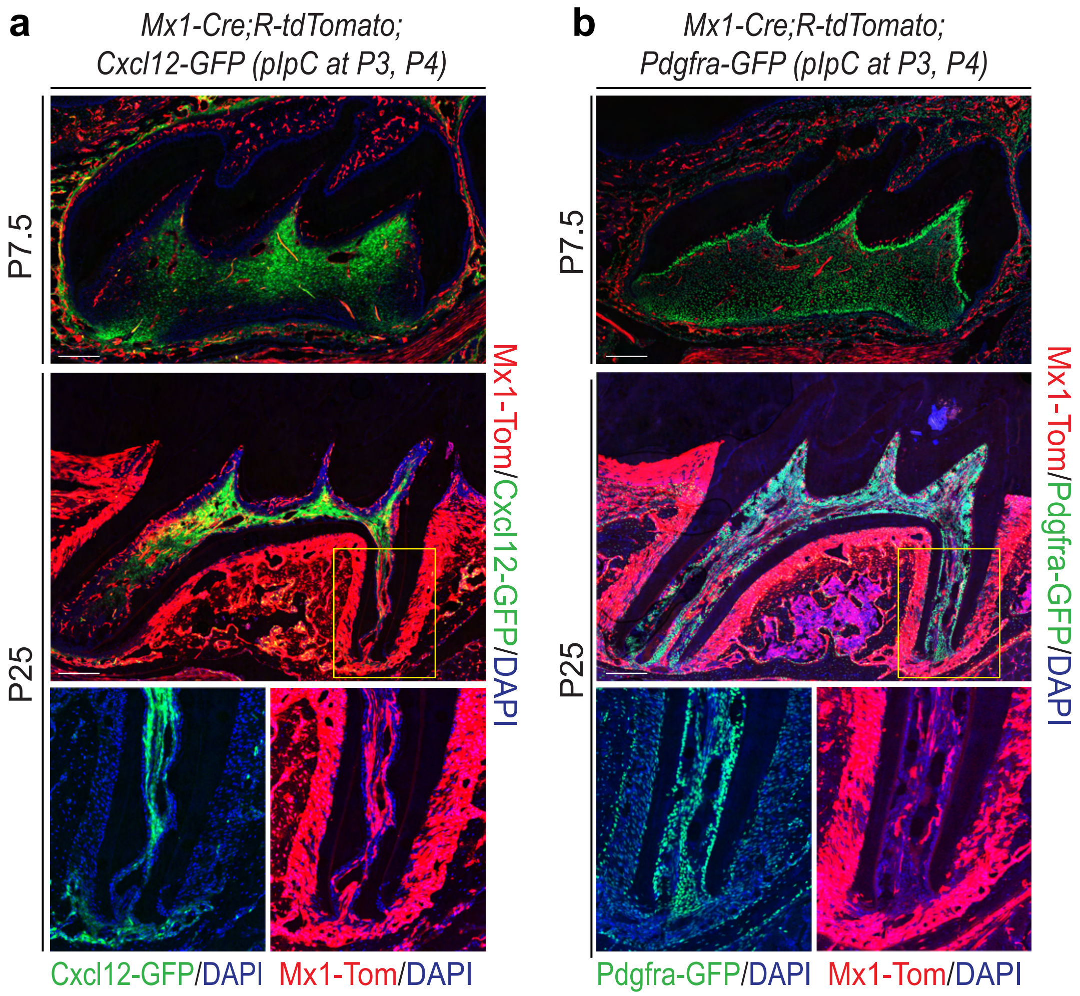

Supplementary Fig. 3: (a) The whole maxillary first molar tooth section of Mx1-Cre;Rosa-tdTomato;Cxcl12-GFP reporter mice at P7.5 and P25, which is enlarged in Figure 2b. (b) The whole maxillary first molar tooth section of Mx1-Cre;Rosa-tdTomato;Pdgfra-H2B-GFP reporter mice at P7.5 and P25, which is enlarged in Figure 2d. The yellow boxes at P25 of (a) and (b) enlarged and split their channel with GFP-DAPI (left) and Tom-DAPI (right). Scale bar: 200 µm.

SUPPLEMENTARY FIGURE 4

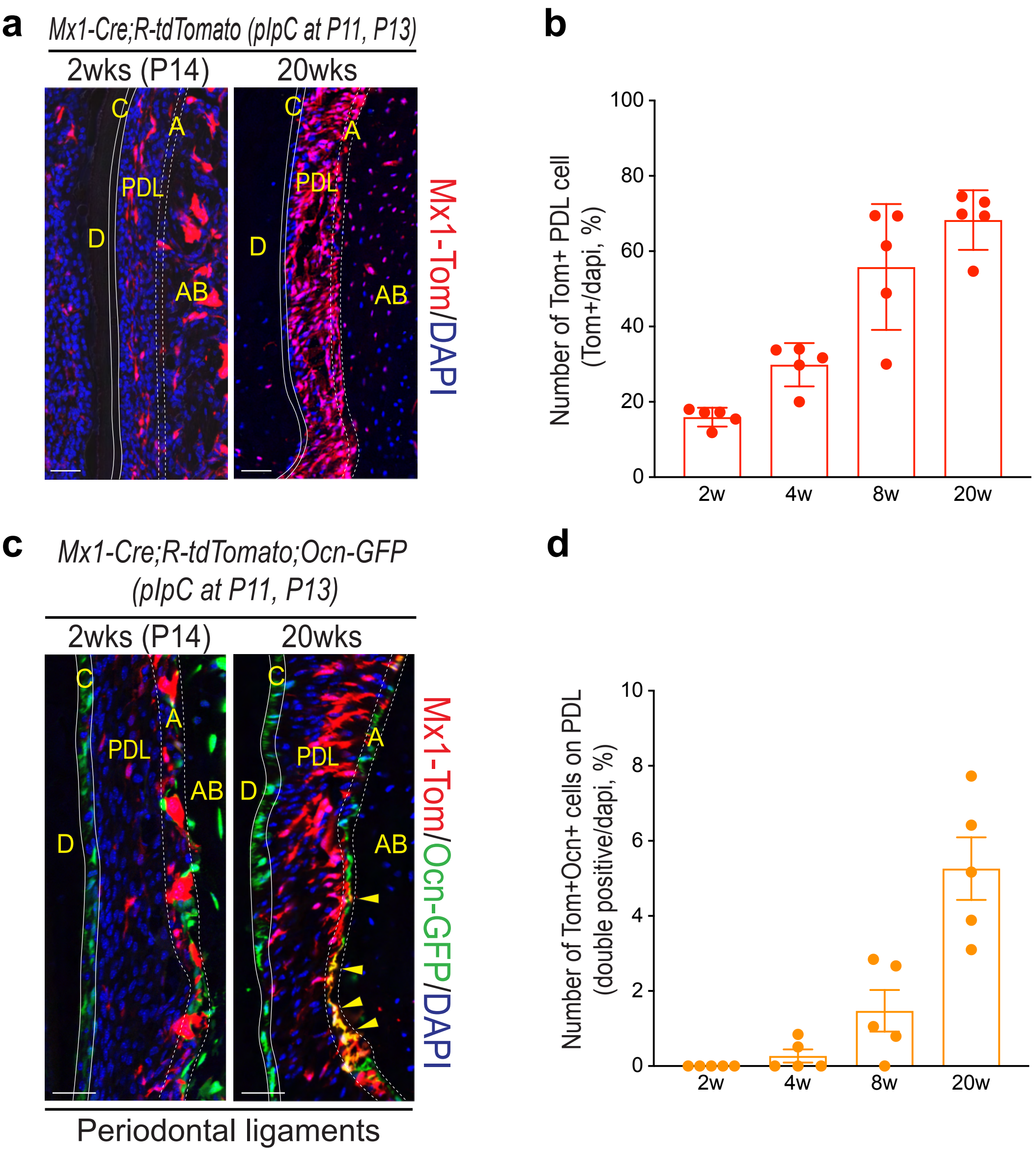

Supplementary Fig. 4: (a) The expression of Mx1+ cells on periodontal ligament for 2 and 20 weeks of age. (b) The numbers of Tom+ cells on PDL were counted by 500 μm^2 of each section (n=5). Results are displayed as mean ± SD. (c) The expression of Mx1+ and Ocn+ cells on periodontal ligament for 2 and 20 weeks of age and their overlap (yellow wedge). (d) The number of Tom^+GFP^-, Tom^-GFP^+, and Tom^+GFP^+ cells within the PDL at different ages were counted based on sections (500 μm^2, n=5). D, Dentin; C, Cementum layer (solid line inside); PDL; Periodontal ligament; A, Alveolar bone layer (dotted line inside); AB, Alveolar bone. Scale bar: 50 μm.

SUPPLEMENTARY FIGURE 5

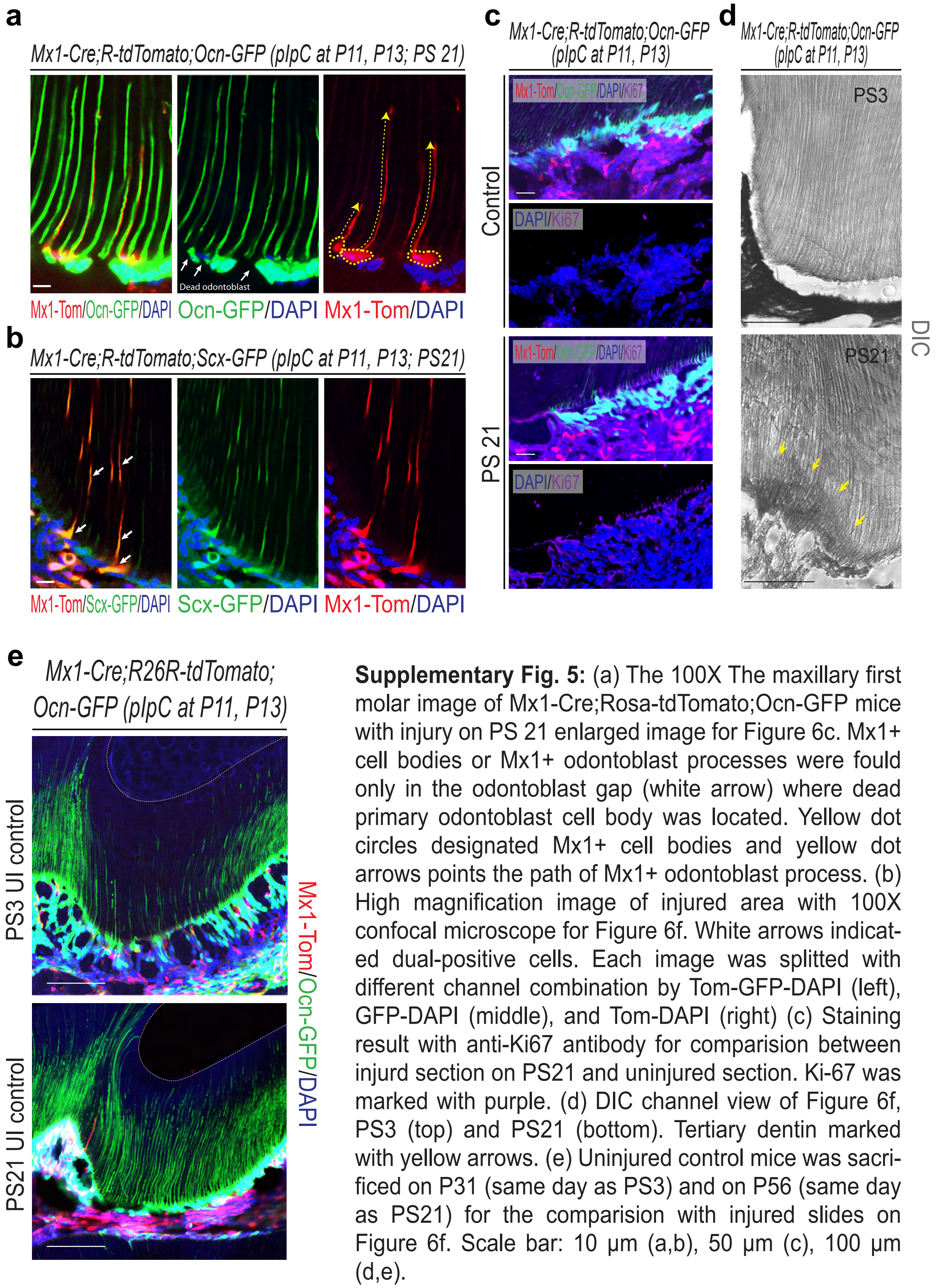

Supplementary Fig. 5: (a) The 100X The maxillary first molar image of Mx1-Cre;Rosa-tdTomato;Ocn-GFP mice with injury on PS 21 enlarged image for Figure 6c. Mx1+ cell bodies or Mx1+ odontoblast processes were fould only in the odontoblast gap (white arrow) where dead primary odontoblast cell body was located. Yellow dot circles designated Mx1+ cell bodies and yellow dot arrows points the path of Mx1+ odontoblast process. (b) High magnification image of injured area with 100X confocal microscope for Figure 6f. White arrows indicated dual-positive cells. Each image was splitted with different channel combination by Tom-GFP-DAPI (left), GFP-DAPI (middle), and Tom-DAPI (right) (c) Staining result with anti-Ki67 antibody for comparision between injurd section on PS21 and uninjured section. Ki-67 was marked with purple. (d) DIC channel view of Figure 6f, PS3 (top) and PS21 (bottom). Tertiary dentin marked with yellow arrows. (e) Uninjured control mice was sacrificed on P31 (same day as PS3) and on P56 (same day as PS21) for the comparision with injured slides on Figure 6f. Scale bar: 10 μm (a,b), 50 μm (c), 100 μm (d,e).

SUPPLEMENTARY FIGURE 6

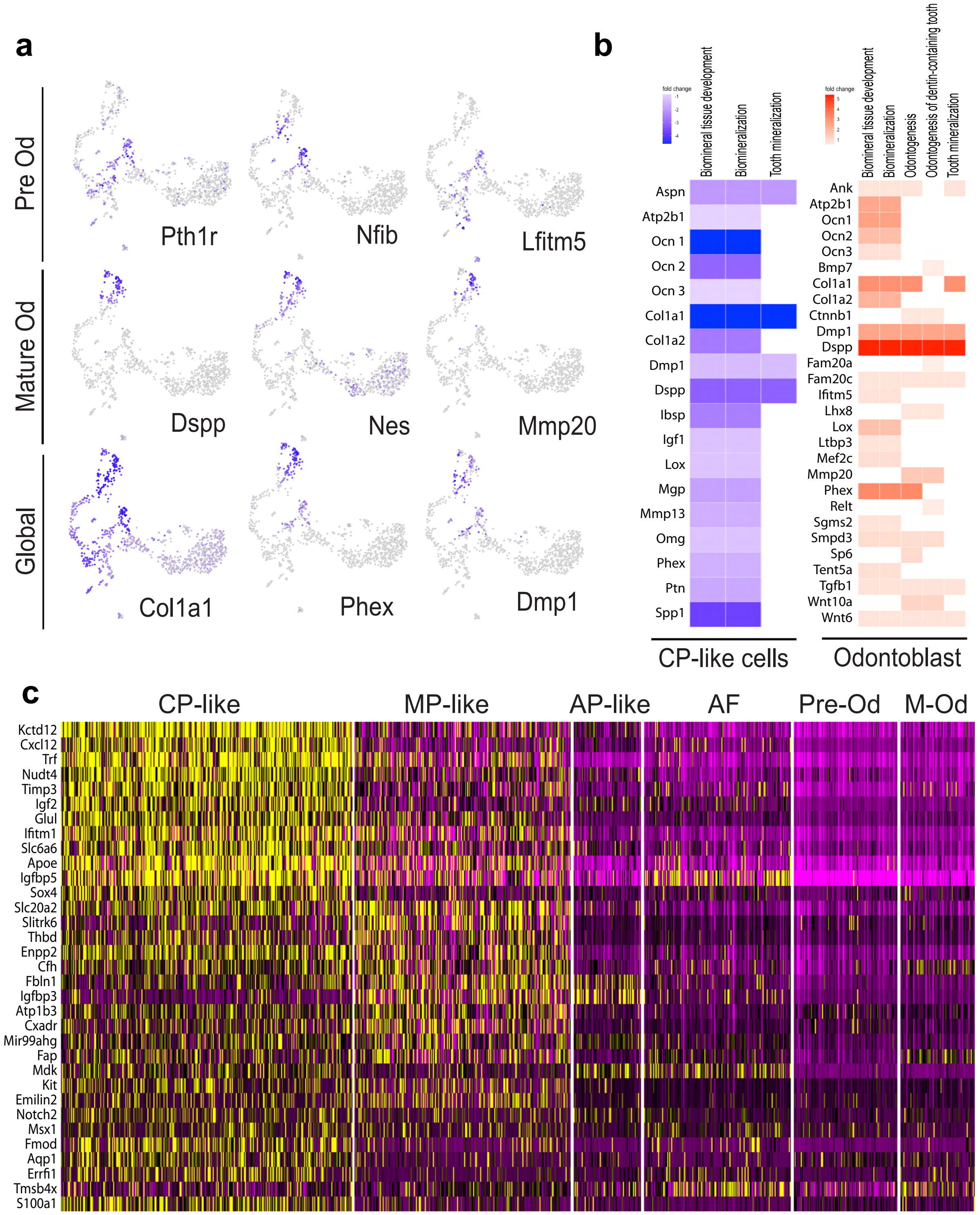

Supplementary Fig. 6: (a) UMAP-based transcriptional plots of odontoblast markers classified based on the degree of their maturation. (b) Gene ontology analysis of tooth mineralization and odontoblast-related genes in the Mx1+ CP-like cluster and odontoblast cluster. (c) The heatmap analysis visually displays a comparison of the expression levels of genes that exhibit high expression in CP-like cells in contrast to other clusters.
